# Vocabulary interventions for children with developmental language disorder: a systematic review

**DOI:** 10.3389/fpsyg.2025.1517311

**Published:** 2025-03-19

**Authors:** Rafiah Ansari, Shula Chiat, Martin Cartwright, Ros Herman

**Affiliations:** ^1^Department of Language and Communication Science, School of Health and Psychological Sciences, City St George's, University of London, London, United Kingdom; ^2^Department of Health Services Research and Management, School of Health and Psychological Sciences, City St George's, University of London, London, United Kingdom

**Keywords:** developmental language disorder, child language acquisition, word learning, vocabulary interventions, systematic review

## Abstract

**Introduction:**

Developmental language disorder (DLD) is a neurodevelopmental condition often characterised by vocabulary difficulties that lead to academic and social challenges. The acquisition of vocabulary is a complex, dynamic process of mapping word sound (phonology) to meaning (semantics) supported by contextual cues; a complexity that vocabulary interventions need to address. To understand the key features and impact of such interventions, a systematic review of word-learning studies involving children aged 5–11 with DLD was conducted.

**Method:**

A structured search covered seven electronic databases for the period 1990–2023. In addition, the reference lists of identified studies were searched manually. Studies were appraised for quality and data was extracted relating to word-learning effectiveness and intervention characteristics. Findings were reported as written summaries and quantitative data ranges.

**Results:**

Sixteen relevant studies were identified with most appraised as medium quality. Interventions tended to be delivered individually in school by speech and language therapists. The most common outcome measure was expressive target-word tests, such as picture naming and word definitions. Interventions explicitly targeting phonological and semantic word features had the most high-quality studies reporting significant vocabulary gain. The inclusion of stories to provide context implicitly during phonological and semantic interventions was beneficial, though stories alone were less effective. Specificity in learning was noted across studies. Gains did not generally transfer to non-targeted words and showed depreciation following therapy. Intervention responses were influenced by children's language profiles. For example, children with more severe language difficulties were less responsive to contextual cues during story reading and were more distracted by extraneous music during multimedia-supported word learning.

**Discussion:**

Whilst the available studies have limitations in range and quality, they do suggest some benefits of combining explicit and implicit vocabulary strategies and considering children's presenting profiles. Implications for practitioners supporting the individual needs of children with DLD are discussed. This includes addressing issues with the generalization and maintenance of vocabulary gains by targeting the most relevant words and encouraging recall and self-management strategies. Further research should explore the influence of home-school carryover.

**Systematic review registration:**

https://www.crd.york.ac.uk/prospero/display_record.php?ID=CRD42022327345, PROSPERO, Reg: CRD42022327345.

## 1 Introduction

Developmental language disorder (DLD) is a condition characterized by significant challenges in daily communication that are unlikely to resolve without specialist intervention (Bishop et al., 2017). It is estimated to affect 7% of children of primary school age, i.e., 5–11-year-olds (Norbury et al., 2016), and can present in isolation, or alongside other neurodevelopmental disorders such as Attention-Deficit/Hyperactivity Disorder (ADHD) and Dyslexia (Bishop et al., 2017).

The term DLD was advocated by Bishop et al. (2017) following a multinational, multidisciplinary consensus study which aimed to align criteria and terminology for children with language difficulties. Contributors to the consensus included speech and language therapists (SLTs), educational psychologists, psychiatrists, pediatricians and specialist teachers. DLD was proposed as a descriptive label focusing on the sustained social and educational impact of language difficulties (Bishop et al., 2017). It served to replace Specific Language Impairment, a term that had been commonly used by clinicians and academics to identify language impairment based on discrepancies between language and intelligence scores (Stark and Tallal, 1981; Tomblin et al., 1997), but which had been criticized for variations in diagnostic threshold and criteria when applied in research and practice (Aram et al., 1993).

Whilst DLD can affect many aspects of language, a restricted vocabulary size and range is among the most common. Almost half of primary-school-aged children with DLD may struggle with vocabulary skills (Rice and Hoffman, 2015). Unsurprisingly, the identification of needs is most common in the primary school years (Lindsay and Strand, 2016), given that this period marks an expected estimated vocabulary growth from 3,000 to 8,000 words (Anglin et al., 1993; Biemiller and Slonim, 2001). This also means that, without intervention, children with DLD are at high risk of falling behind their peers.

Poor vocabulary rarely occurs on its own and is associated with wider language difficulties including issues with grammar and narration (Justice et al., 2018; Khan et al., 2021), whilst also predicting progression in areas of learning such as reading and numeracy, and overall academic attainment (Bleses et al., 2016; Matte-Landry et al., 2020). In addition, vocabulary development trajectories are strongly associated with subsequent behavioral and emotional needs (Westrupp et al., 2020).

### 1.1 Theoretical models

Language acquisition theory and research commonly use a connectionist model of language processing to explain how word learning is a dynamic process of identifying, connecting and mapping spoken sounds (phonology) to their correct meaning (semantics) (Plaut, 1999; Trueswell et al., 2013). It is suggested that children with DLD may struggle with their phonological processing, their semantic processing, and/or connecting the two during word learning, leading to issues with understanding (receptive) and/or use (expressive) of words (Best et al., 2015; Chiat, 2001).

Context plays a crucial role in word learning, providing social, perceptual, cognitive and linguistic signals to help connect sounds to meaning (Monaghan, 2017; Pomper, 2020). A cross-situational learning model has been proposed to describe how learners use contextual information from multiple situations to track the co-occurrence of word sounds and their meanings and to resolve ambiguity in sound-meaning associations (Roembke et al., 2023; Hartley et al., 2020). There are indications of restricted capacity in using this contextual inferencing to support language processing in children with DLD (Broedelet et al., 2023; McGregor et al., 2022).

### 1.2 Intervention approaches

Interventions for children with DLD are predominantly led by SLTs, who deliver evidence-based therapy in conjunction with parents, educators, and partner professionals such as educational psychologists. Therapy tends to occur in the home or clinic when children are younger and then usually moves to mainstream or specialist school settings. Language goals can vary but often include those relating to the child's interest, school curriculum and family routine (Dennis et al., 2017).

Empirical coverage of vocabulary interventions for children with DLD tend to focus on strategies that elaborate and connect the sound and meaning components of words (Steele and Mills, 2011). The goal is to increase children's accuracy in the understanding and use of word sound features (e.g., initial sounds, syllables, and rhymes) and word meaning features (e.g., function, location, category, attributes). Activities are explicit, meaning the child is actively taught word features through tasks involving imitation, repetition, feedback, and recall; written words and pictures often serve as prompts (for example activities see Parsons et al., 2005). The rationale is that by directly targeting the sound and meaning of words, children with DLD are supported to undertake the phonological and semantic integration required for word learning, a method that is in line with the connectionist model of language processing (Plaut, 1999; Trueswell et al., 2013).

An alternative approach to vocabulary interventions for children with DLD is the use of implicit, incidental strategies to provide contextual cues to support word learning, a method more aligned to a cross-situational learning model (Broedelet et al., 2023). Presenting target words in a narrative using story-based activities is an example of a context-based intervention for vocabulary enrichment (see Nash and Donaldson, 2005). The content of the stories can facilitate vocabulary learning by providing information regarding word definitions as well as examples of how the word can be used outside of therapy (Marks and Stokes, 2010). In addition, the grammatical structures surrounding the target word when presented in a narrative can provide important clues around word meaning. This is referred to as syntactic bootstrapping, a process where the syntactic frame and morphological markers associated with a novel word help to determine the meaning of the word (Rice et al., 2000).

Whilst explicit semantic-phonological interventions and more indirect contextual interventions have differing theoretical basis and strategies, they can be considered complementary approaches to supporting vocabulary skills in children with DLD. A survey of SLT vocabulary-intervention practice for school-aged children (Steele, 2020, US survey with 357 respondents) identified that therapists most frequently used direct explicit strategies (endorsed by 91.3% of respondents) with the next most common approach being context-based strategies (endorsed by 79.7% of respondents). It is worth noting that therapy decisions were predominantly driven by professional experience rather than a consideration of research findings, a pattern that has emerged in other studies of SLT vocabulary-intervention practice (Justice et al., 2014; Marante and Hall-Mills, 2024). The reason appears to be difficulty applying research into practice given the heterogeneity of the DLD population. The nature, extent, and implications of language difficulties may vary not only between children but also within a child due to differential influence of internal physiological and psychological and external social and environmental factors (Law et al., 2022). This highlights the need for research that helps practitioners understand not only the outcomes of vocabulary-intervention studies, but also the key characteristics of the interventions to help incorporate evidence-based strategies into individualized support for children with DLD.

### 1.3 Intervention research

Whilst there are several systematic reviews that have synthesized the evidence base for vocabulary interventions for children with DLD, only two have covered the primary school period, i.e., 5–11 years. Cirrin and Gillam (2008) analyzed 21 peer-reviewed studies of language interventions for children aged 5–18 years. Of the studies reviewed by Cirrin and Gillam (2008) only one reported the significance and size of vocabulary intervention effects for children in the primary school years (Wing, 1990, *n* = 10, age 5; 11–7; 01). This was a non-randomized matched-group comparison of a specialist-school-based SLT-delivered intervention targeting word phonology (picture cards to support the understanding and use of initial sounds, syllables, and rhymes for target words) versus a semantic approach (picture cards to support understanding and use of word category, function, and attributes). Only the phonological approach led to a significant vocabulary gain with a moderate effect size (*p* < 0.05, *d* = 0.7) as measured using a standardized expressive vocabulary test.

The second systematic review (Rinaldi et al., 2021) focused on randomized controlled language intervention trials for children aged 3–8 years. Only one vocabulary intervention paper was identified (Smeets et al., 2014), comparing target words implicitly presented in picture or video animation narrated e-stories. The children, who were based in specialist schools, were encouraged to view the e-stories independently. Two studies were conducted, both adopting a randomized alternating treatment crossover design. The first study (*n* = 28, age 5; 0–6; 8) found significantly greater gain on an expressive sentence completion test for words exposed through e-stories compared to non-targeted words (*p* < 0.001, *d* = 1.54, large effect size) with greater gain for picture e-stories without background music than animated e-stories with background music (*p* < 0.01, *d* = 0.48, medium effect). The second study found that, in the absence of background music, vocabulary gain did not differ between e-story types (*n* = 21, age 5; 0–7; 6).

The above studies are informative, however, having only two vocabulary-intervention papers for primary-school aged children with DLD that have been reviewed as empirically robust, limits the evidence-base for practitioners, researchers and academics. Given that one review of studies is over a decade old, an update is warranted to cover subsequent relevant studies. While the other review, by considering only randomized control trials and having an upper age cut-off of 8 years, may have excluded studies of relevance. There is therefore a need for a systematic review that explores the fundamental features and impact of vocabulary interventions in studies that spans the primary school years and have sufficient methodological rigor to be of empirical value. In keeping with this, the current review addresses the following research question:

What are the key characteristics of vocabulary interventions for primary-school-aged children with DLD and their influence on word-learning outcomes?

## 2 Method

A systematic review of word-learning studies was undertaken to identify the core components of vocabulary interventions, the intervention effects, and the study design that generated the outcomes. The review was completed according to the Preferred Reporting Items for Systematic Reviews and Meta-Analyses (PRISMA, Page et al., 2021). The review is registered in PROSPERO (Patel et al., 2022, Reg: CRD42022327345; https://www.crd.york.ac.uk/prospero/display_record.php?ID=CRD42022327345).

### 2.1 Eligibility

Eligible studies included children aged 5; 0–11; 11 with diagnoses of DLD or equivalent according to the criteria in Bishop et al. (2017) as described below:

- Children presenting with difficulty producing or understanding language that affected everyday functioning (everyday social interactions or educational progress).
- If the child was multilingual, then the difficulties presented in all languages.
- The presentation had to be suggestive of poor prognosis with difficulties emerging in the course of development. This is based on research evidence indicating that language problems apparent from preschool years that are still evident at 5 years and over are likely to persist (Stothard et al., 1998).
- The language difficulties could not be acquired or associated with a known biomedical cause. Children with a language difficulty secondary to a biomedical condition, where language needs occur as part of more complex impairments patterns, were excluded on the basis of requiring specialized intervention. Differentiating conditions include brain injury, neurodegenerative conditions, cerebral palsy, sensori-neural hearing loss, and genetic conditions such as Down syndrome. In line with recommendations from Bishop et al. (2017), autism and intellectual disability were considered differentiating conditions in this review as they are commonly linked to genetic or neurological causes.
- Children with a language and co-occurring cognitive, sensori-motor or behavioral disorders, which may affect pattern of language impairment and intervention response but where causal relation is unclear, were considered to meet the criteria for DLD. These co-occurring disorders include attentional problems, motor problems, literacy problems, speech problems, limitations of behavior and emotions.

While DLD is the empirically-advocated term for the population in this review (Royal College of Speech Language Therapists: RCSLT, 2021; McGregor et al., 2020), the search strategy also accounted for overlapping academic and clinical diagnostic labels including (specific) language impairment, language delay and language difficulties (Green, 2020; Georgan and Hogan, 2019).

The study designs considered were randomized control trials (RCTs), non-randomized controlled studies and pre-post comparisons. Single-subject designs were only considered if outcomes were measured at multiple timepoints. Studies had to have reported on or had sufficient data to calculate the significance of the change with or without effect size values.

Any intervention aimed at improving vocabulary (with or without standard treatment) was considered for the review as well as any comparator. Studies were required to have a vocabulary measure as a primary outcome. Secondary measures, such as grammar and literacy outcomes, were recorded but were not a key focus as it would be difficult to infer direct causal effects.

Only English language publications were reviewed, as time and resources were not available for reliable translation, though this is acknowledged as a limitation. Only studies reported on or after 1990 were considered based on the date of the earliest vocabulary intervention study identified in previous comparable systematic reviews (Cirrin and Gillam, 2008).

### 2.2 Search strategy

To identify relevant studies, searches were conducted for published trials between 1990 and 2023 in PubMed, CINAHL, PsycINFO, the Cochrane Library, and ERIC. In addition, unpublished literature between 1990 and 2023 was searched using SCOPUS and Open Dissertations.

For each database a search strategy was developed by considering MESH and free terms which covered the following: (Language AND (disorder^*^ OR impair^*^ OR delay^*^ OR difficult^*^)) AND (child^*^ OR infant^*^ OR P?ediatric^*^) AND (vocabulary OR word^*^) AND (therap^*^ OR intervention^*^ OR instruction^*^ OR treatment^*^ OR teaching OR learning OR support^*^). In addition, manual searches of reference lists of identified studies were conducted.

### 2.3 Manual data management

Study data were transferred for refinement and coding using EPPI-Reviewer systematic review software (V4 https://eppi.ioe.ac.uk/cms/Default.aspx?alias=eppi.ioe.ac.uk/cms/er4).

### 2.4 Data selection

The primary author reviewed selected papers, screened titles and abstracts to remove ineligible studies, then conducted a full text review of the remaining articles to identify eligible studies. A third of selected titles and abstracts, as well as all selected full texts, were reviewed for consensus by a secondary rater. To quantify the level of agreement between the two raters, the Cohen's Kappa measure of inter-rater reliability was used. An agreement value of 0.82 was achieved for the selection of titles and abstracts and then again for the selection of full texts. This was acceptable as a value >0.8 is considered satisfactory (Pérez et al., 2020).

### 2.5 Data extraction

Data extraction was guided by the Cochrane Data Extraction Form for RCTs and non-RCTs (Wilson, 2016) and the Template for Intervention Description and Replication (TIDieR; Hoffmann et al., 2014). Collectively, this provided information on the studies investigating the interventions (author, year, country, design, participants, sample sizes, target words, outcome measures, follow up) and the characteristics of the interventions (type, dosage, provider, mode, location).

### 2.6 Quality appraisal

The selected studies were appraised to assess their methodological quality and the extent to which each study had addressed the possibility of bias in its design, conduct and analysis. The Joanna Briggs Institute critical appraisal checklists (Tufanaru et al., 2020) were used to assess quality as they can be applied to multiple study designs and have precedent for use with DLD populations (Alduais et al., 2022; Wanicharoen and Boonrod, 2024; Zupan et al., 2022). The primary author and a second rater independently appraised each included study and reached full consensus.

The checklists assessed areas relating to selection bias, study design, confounders, and data collection methods with a choice of yes/no/unclear/not applicable responses. Full details of the checklist and results are available in Appendix A. Raw scores were calculated for each selected study by dividing the number of positive responses by the total number of applicable statements in the critical checklists, these were then converted to percentage scores. Studies with percentage scores of < 49% were classed as low quality with high risk of bias, studies between 50% and 79% as medium quality, and studies ≥80% as high in quality with low risk of bias. This classification has been used in prior systematic reviews (e.g., Zupan et al., 2022).

As per recommended guidelines (Tufanaru et al., 2020), studies of low-quality were included in the qualitative reporting to provide a complete view of the evidence available to inform the review question. However, findings from low-quality studies were omitted from the quantitative synthesis to minimize the impact of study biases when pooling numerical data.

## 3 Results

This section details the word-learning studies identified in the review and provides a narrative summary of intervention characteristics. This is followed by a quantitative synthesis of studies with comparable design in order to synthesize intervention outcomes by prominent intervention characteristics.

### 3.1 Identification of studies

**Figure 1:**
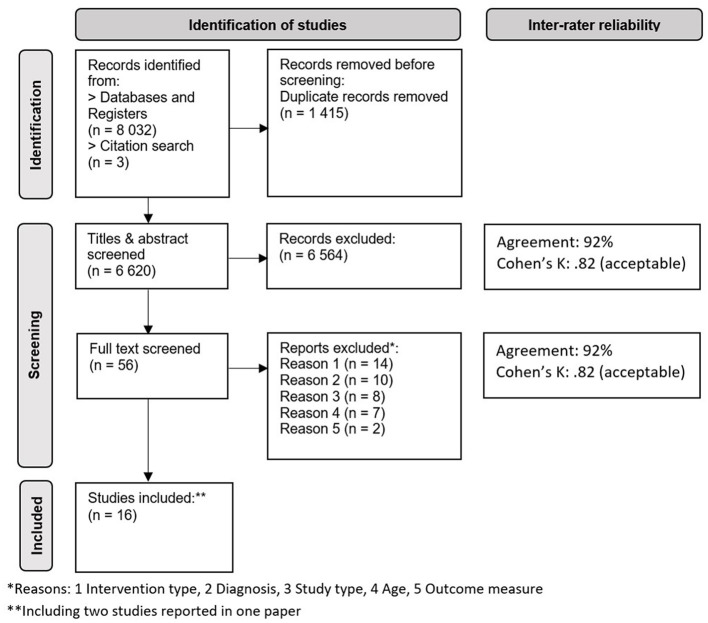
Flow diagram of the article screening process (based on PRISMA guidelines, Page et al., 2021).

The search was completed on 1st October 2023 and database alerts were set up to signpost any new studies that were subsequently published (none were identified). In total, the search yielded 16 studies meeting the eligibility criteria, 12 with interventions delivered in English (UK, 7 studies; US, 4 studies; New Zealand, 1 study), two in Dutch (set in the Netherlands), one in French (set in Switzerland), and one in German (set in Germany), providing collective data for 288 participants aged 5; 0–11; 11 (167 males, 78 females, 43 unknown). A PRISMA flow diagram of the article screening process is presented in Figure 1.

### 3.2 Study data

A summary of data from the selected studies is presented in Tables 1–4. Studies in this review were aligned to four intervention approach types to enable better synthesis and analysis of findings: interventions that explicitly focused on phonological and semantic word features to support learning (Table 1), interventions utilizing written and/or verbal stories to target word learning (Table 2), use of stories and phonological/semantic approaches combined (Table 3), and interventions targeting orthography (spellings) to support word learning (Table 4).

**Table 1:** Summary of reviewed studies – phonological and/or semantic vocabulary intervention approaches.

**Authors, year, country**	**Design**	**Participants**	**Intervention**	**Dosage**	**Delivery**	**Outcome measure**	**Results**	**Follow-up**	JBI quality rating^*^
Wing (1990), US	Non-randomized matched group comparison: 5 children per group	8 males & 2 females with existing diagnosis of LI (age 5; 11–7; 1)	Phonological versus semantic approach	30 × 25 min sessions over 2.5 months per group Total: 750 min/12.5 h Number of target words unclear	**Provider**: School SLT **Mode**: Face-to-face group sessions in English **Location**: Room in specialist school	Within group, pre-post comparison using the standardized Test of Word Finding (German, 1986), a picture-naming test of expressive vocabulary	•Significant gain with phonological therapy (*p* < 0.05, *d* = 0.7, moderate effect) •Gain with semantic therapy was not significant	Not measured	Low quality
Wright (1993), UK	Matched no-treatment control group: 2 males & 2 females with SLI (aged 7; 9–8; 5)	2 males & 2 females with existing diagnosis of SLI (age 7; 6–8; 8)	Combined phonological & semantic approach	18 × 20 min sessions over 4 weeks Total: 360 min/6 h90 target words per child, each presented once	**Provider**: School SLT **Mode**: Face-to-face group sessions in English **Location**: Room in specialist school	Within group, pre-post comparison using a researcher-created picture-naming test of target words to assess expressive vocabulary	•Significant gain for target words (*p* < 0.01) & untargeted control words (*p* < 0.05) •No significant change for control group on any measure Effect sizes not reported	Loss in gains at 1-month follow up	Medium quality
Parsons et al. (2005), UK	Pre-post comparison	2 males with existing diagnosis of SLI (age 8; 10–9; 5)	Combined phonological & semantic approach	18 × 25–35 min sessions. 2–3 sessions a week over 8 weeks Total: 450-630 min/7.5–10.5 h18 target words, single presentation in therapy with home-school reinforcement	**Provider**: Healthcare SLT with carer follow-up at home & teaching staff follow-up in class **Mode**: Face-to-face individual sessions in English **Location**: Room in mainstream school	1. Individual pre-post comparison using a researcher-created test which matched targeted words with pictures/synonyms to assess receptive vocabulary 2. Individual pre-post comparison using standardized vocabulary measures: British Picture Vocabulary Scale (Dunn et al., 1982) to assess receptive vocabulary & Test of Word Finding (German, 1989) to assess expressive vocabulary	1. Target word tests •Child A. Significantly greater gain for target words than control words (*p* < 0.01) •Child B. Significantly greater gain for target words than control words (*p* < 0.001) Effect sizes not reported 2. Standardized tests. No change for Child A or B	Not measured	Medium quality
Zens et al. (2009), New Zealand	Randomized alternating treatment crossover design, no washout time in-between	19 children with existing diagnosis of SLI (age 6; 2–8; 3, gender unknown)	Phonological/semantic/combined approach + Ongoing specialist support for 10 children (specifics unclear)	12 h of one intervention over 6 weeks (2 × 1 h weekly) Followed by 12 h alternate intervention over 6 weeks (2 × 1 h weekly) Total: 720 min/12 h 27 target words, multiple presentations	**Provider**: University-affiliated SLT **Mode**: Face-to-face group sessions in English **Location**: Room in mainstream school	1. Within group, pre-post-test comparison using the standardized Test of Language Development 3rd Ed (Newcomer and Hammil, 1997) & a researcher-created categories test. Raw scores were combined to provide an expressive vocabulary score 2. Within group, pre-post-test comparison using the non-standardized Phonological Awareness Probes (Stahl and Murray, 1994): phoneme blending, isolation, segmentation & deletion	1. Expressive vocabulary tests •Significant gain with phonological therapy (*p* = 0.001, f = 0.65, large effect) •Significant gain with phonological + semantic therapy (*p* = 0.001, *f* = 0.65, large effect) •Significant gains with semantic therapy (*p* = 0.004, *f* = 0.62, large effect) •Significant gain with semantic + phonological therapy (*p* = 0.004, *f* = 0.62, large effect) 2. Phonological awareness tests •Significant gain with phonological therapy (*p* < 0.001, *f* = 1.06, large effect) •Significant gain with phonological + semantic therapy (*p* < 0.001, *f* = 1.06, large effect)	Not measured	Medium quality
							•Significant gain with semantic + phonological therapy *(p* < 0.001, *f* = 1, large effect) •Gain with semantic therapy was not significant		
Motsch and Marks (2015), Germany	Randomized control trial. Control: 55 males & 24 females with SLI (mean age 9;6, SD 0.27)	53 males & 25 females with existing diagnosis of SLI (mean age 9;6, SD 0.16) German-speaking. 38 children received group therapy (2 children per group). 40 received individual therapy	Combined phonological & semantic approach + Variety of ongoing individual & group SLT/teacher language support (specifics unclear, however statistical significance of intervention gains maintained when this additional-support cohort removed)	20 sessions once per week over 5 months. 45 min group sessions & 30 min individual sessions Total: Individual therapy – 600 min/10 h Group therapy – 900 min/15 h Number of target words unclear	**Provider**: SLTs with home-school follow-up **Mode**: Face-to-face individual versus group sessions in German **Location**: Room in special schools	Within and between group pre-test to 4-month follow-up comparison using a range of standardized language tests in German (post-test scores not reported): 1. WWT 6–10 (Glück, 2011). A picture- naming test of expressive vocabulary 2. P-ITPA - Vocabulary subtest (Esser et al., 2010). A sentence-completion test to assess expressive vocabulary 3. P-ITPA – Analogies subtest (Esser et al., 2010). An analogy-generation test to assess expressive vocabulary 4. SET 5–10 (Petermann, 2010). A sentence comprehension to assess receptive syntax	Within group 1. Picture naming test of expressive vocabulary •Significant gain with group therapy (*p* < 0.001, *d* = 0.73, large effect) •Significant gain with individual therapy (*p* < 0.001, *d* = 0.54, medium effect) 2. Sentence completion test of expressive vocabulary •Significant gain with group therapy (*p* = 0.004, *d* = 0.38, medium effect) •Gains did not reach significance with individual therapy 3. Analogies test of expressive vocabulary. No gains reached significance 4. Sentence comprehension test •Significant gain with group therapy (*p* = 0.02, *d* = 0.41, medium effect) •Significant gain with individual therapy (*p* < 0.001, *d* = 0.57, medium effect) Between group 1. Picture naming test. Gain for group therapy significantly greater than control group gain (*p* = 0.039) 2. Sentence completion test. No significant between-group difference 3. Analogies test. Gain for individual therapy significantly greater than control group gain (*p* = 0.01) 4. Sentence comprehension test. Gain for individual therapy significantly greater than control group gain (*p* = 0.039)	Change from post-test to follow-up not reported, only pre-test to 4-month follow-up	High quality
Best et al. (2018), UK	Randomized control trial Control: 5 males & 4 females with DLD (aged 6; 3–8; 7)	6 males & 5 females diagnosed with DLD as part of the study (age 6; 0–7; 8)	Word webs for combined phonological & semantic approach No other intervention accessed	Weekly 30 min sessions for 6 weeks Total: 180 min/3 h 25 target words, multiple presentation	**Provider**: University SLT **Mode**: Face-to-face individual sessions in English **Location**: Mostly room in mainstream school	Within and between group pre-post comparison using a researcher-created picture-naming test of target words to assess expressive vocabulary	Between group •Significantly greater gain for therapy group than control group on target-words (*p* < 0.0001, *d* = 2.30, large effect), no significant difference on control words	Not measured	High quality
Best et al. (2021), UK	Randomized alternating treatment crossover design with 6-week washout	12 males & 8 females diagnosed with DLD as part of the study (age 6; 4–8; 8)	Word webs for phonological versus semantic approach No other intervention accessed	Weekly 30 min sessions for 6 weeks per approach. Total: 180 min/3h 50 target words, 25 per approach, multiple presentation	**Provider**: University SLT **Mode**: Face-to-face individual sessions in English **Location**: Mostly room in mainstream school	1. Within group pre-post comparison using a researcher-created picture-naming test of target words to assess expressive vocabulary 2. Outcomes according to language profile	1. Within group •Significantly greater target-word gain for semantic therapy than phonological therapy (*p* = 0.014, *d* = 0.489, medium effect) •No significant order effects or change in control words 2. Outcomes according to language profile •Children with semantic & phonological needs (*n* = 11): 3 children showed significant gain from semantic intervention only, 2 children from phonological intervention only, 5 children from both interventions, 1 child showed no significant gain from either intervention •Semantic needs (*n* = 6): 4 children showed significant gain from semantic intervention only, 2 children showed no significant gain from either intervention •Phonological needs (*n* = 3): 2 children showed significant gain from phonological intervention only, 1 child showed no significant gain from either intervention	Loss in gains at 6-week follow up	High quality
Ardanouy et al. (2023), Switzerland	Pre-post comparison	8 French-speaking children with existing diagnosis of DLD (age 6–10 years, gender unknown)	Combined phonological & semantic approach with context cues No other intervention accessed	5 months of 45 min session per week. 4 sessions per theme covering 4 themes (sports, animals, vegetables, & school materials) Total: 840 min/14 h 60 target words, 15 per category, multiple presentation	**Provider**: University SLT supported by Educational Psychologists **Mode**: Face-to-face group sessions in French **Location**: Specialist clinic	Within group and individual pre-post comparison using a researcher-created picture-naming test of target words to assess expressive vocabulary. Target words were grouped by category	Within group •Veg: Significantly greater gain for target words than control words (*p* = 0.01, *r* = 0.89, large effect) •Animals: Significantly greater gain for target words than control words (*p* = 0.01, *r* = 0.89, large effect) •Sports: Significantly greater gain for target words than control words (*p* = 0.01, *r* = 0.89, large effect) •School: Significantly greater gain for target words than control words (*p* = 0.02, *r* = 0.84, large effect) Individual level •Veg: Significant gain for 6 out of 8 children (*p* < 0.05) •Animals: Significant gain for all children (*p* < 0.05) •Sports: Significant gain for 7 out of 8 children (*p* < 0.05) •School theme: Significant gain for 4 out of 8 children (*p* < 0.05) •Control words: no significant change	Veg: no change at 1.5-month follow-up Animals: no change at 3-month follow-up Sports: loss in gains at 4.5-month follow-up	Medium quality

^*^Based on JBI quality appraisal rating (see Appendix A).

LI, Language Impairment; SLI, Specific Language Impairment; DLD, Developmental Language Disorder.

**Table 2:** Summary of reviewed studies – story-based vocabulary intervention approaches.

**Authors, year, country**	**Design**	**Participants**	**Intervention**	**Dosage**	**Delivery**	**Outcome measure**	**Results**	**Follow-up**	JBI quality rating^*^
Nash and Donaldson (2005), UK	Non-randomized alternating treatment crossover design with 1-week washout	13 males & 3 females with existing diagnosis of SLI (age 5; 5–9; 0)	Exposure to spoken stories + Corresponding picture books Versus Explicit semantic approach	Two 20–30 min sessions over 2 consecutive days for each learning context Total: 40–60 min 8 target words per child, 4 per approach, multiple presentations	**Provider:** University SLT **Mode:** Explicit teaching: Face-to-face individual sessions with SLT Incidental: Playing of pre-recorded story by SLT **Location:** Mostly room in mainstream school	Within group comparison of gain between first and second therapy session, and between group comparison of final scores, using a range of researcher-created target-word tests to assess expressive & receptive vocabulary. 1. Picture naming test of target words (expressive vocabulary) 2. Target word definition test (expressive vocabulary) 3. Spoken word to picture matching – match target word to 1 of 4 pictures (receptive vocabulary) 4. Spoken word recognition test – correct pronunciation of target word from choice of 4 (receptive vocabulary) 5. Meaning recognition test – Y/N allocation to given category & attribute (receptive vocabulary)	Within group 1. Picture naming test • Significant gain with story exposure (*p* < 0.01) • Significant gain with semantic therapy (*p* < 0.01) 2. Word definition test • Significant gain with story exposure (*p* < 0.05) • Significant gain with semantic therapy (*p* < 0.01) 3. Spoken word to picture matching • No significant change with story exposure • Significant gain with semantic therapy (*p* < 0.01) 4. Spoken word recognition. • Significant gain with story exposure (*p* < 0.001) • Significant gain with semantic therapy (*p* < 0.01) 5. Meaning recognition • Significant gain with story exposure (*p* < 0.05) • No significant change with semantic therapy Between group Overall gain from semantic therapy significantly greater than story exposure for the Word Definition test (*p* < 0.05) and the Meaning Recognition test (*p* < 0.05). All other between group comparisons were non-significant Effect sizes not reported. No order effect analysis	Not measured	Medium quality
Smeets et al. (2014), Study 1, Netherlands	Randomized alternating treatment crossover design with no washout	24 males & 5 females with existing diagnosis of SLI (age 5; 0–6; 8)	Exposure to narrated e-stories with pictures (no background sound) versus videos (with background sounds)	Each approach presented across 8 sessions over 4 weeks in random order. Session lengths unknown 28 target words, 14 per approach, multiple presentations	**Provider:** Academics in psychology **Mode:** Individual sessions in Dutch using headphones & television screen **Location:** Room in specialist school	Between group comparison of pre-post gains using a researcher-created sentence completion test of target words to assess expressive vocabulary	•Target-word gain significantly greater for picture e-books than with video e-books (*p* < 0.01, *d* = 0.48, medium effect) •Target-word gain significantly greater for e-books (picture & video condition combined) than control words (*p* < 0.001, *d* = 1.54, large effect)	Not measured	Medium quality
Smeets et al. (2014), Study 2, Netherlands	Randomized alternating treatment crossover design with no washout	13 males & 10 females with existing diagnosis of SLI (age 5; 0–7; 6)	Exposure to narrated e-storybooks with picture/video with/without background sounds	Each approach presented across 16 sessions over 8 weeks in random order. Session lengths unknown 72 target words, 18 per approach, multiple presentations	**Provider:** Academics in psychology **Mode:** Individual sessions in Dutch using headphones & television screen **Location:** Room in specialist school	Between group comparison of pre-post gains using a researcher-created sentence completion test of target words to assess expressive vocabulary	•No significant differences in target-word scores found between the intervention groups •A significant correlation was found between increased language severity and negative influence of background sound (*p* < 0.05, *d* = 0.43, medium effect)	Not measured	Medium quality

^*^Based on JBI quality appraisal rating (see Appendix A).

SLI, Specific Language Impairment.

**Table 3:** Summary of reviewed studies – story-based with semantic and/or phonological vocabulary intervention approaches.

**Authors, year, country**	**Design**	**Participants**	**Intervention**	**Dosage**	**Delivery**	**Outcome measures**	**Results**	**Follow-up**	JBI quality rating^*^
Marks and Stokes (2010), UK	Pre-post comparison	1 male with LI (aged 8; 1)	Story read to child + semantic approach No other intervention accessed	8 × 50–60 min sessions, over 3 weeks. Total: 400–480 min/6 h 40 min–8 h 30 target words across 4 stories, multiple presentations	**Provider**: Healthcare SLT **Mode**: Face-to-face individual sessions in English **Location**: Room in mainstream school	Within group, pre-post comparison using two researcher-created tests of target words to assess vocabulary: 1. Picture naming test of target words (expressive vocabulary) 2. Spoken word to picture matching (receptive vocabulary)	1. Picture naming test •Significant gain with target words (*p* < 0.001) •No significant changes with control words 2. Spoken word to picture matching •Significant gain with target words (*p* = 0.016) •No significant changes with control words Effect sizes not reported	Loss in gains at 8-month follow up	Medium quality
Steele et al. (2013), US	Randomized alternating treatment crossover design with no washout in-between	10 males & 2 females with existing diagnosis of LI (Mean age 10; 3, SD: 9.32 months)	Child-read story + Phonological and/or semantic approach	One session per condition, length and frequency unknown 15 target words per child, 5 per condition, multiple presentations	**Provider**: University SLT & SLT students **Mode**: Face-to-face individual sessions in English **Location**: Room in mainstream school	Within group, pre-post comparison using a researcher-created target-word definition test to assess expressive vocabulary	•Significantly greater target-word gain for story + phonological + semantic therapy (combined) compared to the control condition (*p* = 0.028) •Significantly greater target-word gain for story + semantic therapy compared to the control condition (*p* = 0.002) •No significant difference between story + phonological therapy compared to the control condition Effect sizes not reported. No order effect analysis	Not measured	Medium quality
Lowman and Dressler (2016), US	Randomized alternating treatment crossover design with no washout time in-between	18 children with existing diagnosis of SLI (age 10; 0–11; 11, gender unknown)	Child-read storybooks + Phonological, semantic & syntactic word cues via an iPod Versus Story reading Ongoing language support continued for both conditions (specifics unclear)	Eight 15-min video viewing sessions, over 4 weeks (2 sessions a week) Total: 120 min/2 h Plus, reading time (not measured) 24 target words, 12 per condition, multiple presentations	**Provider:** Self-directed viewing of iPods/book reading with University-affiliated SLT/SLT students supporting as required **Mode**: Technology vs book **Location**: Room in mainstream school	Between group comparison of pre-post gains using a range of researcher-created target-word tests of expressive & receptive vocabulary 1. Word definition test (expressive vocabulary) 2. Semantic recognition test (receptive vocabulary) 3. Definition selection test (receptive vocabulary) 4. Sentence generation test (expressive syntax) 5. Syntactic recognition test (receptive syntax)	1. Word definition test Significantly greater target-word gain for combined therapy compared to story-reading alone (*p* < 0.05, *f*^2^ = 0.13, small effect) 2. Semantic recognition test Significantly greater target-word gain for combined therapy compared to story-reading alone (*p* < 0.01, *f*^2^ = 0.11, small effect) 3. Definition selection test Significantly greater target-word gain for combined therapy compared to story-reading alone (*p* < 0.01, *f*^2^ = 0.46, large effect) 4. Sentence generation test Significantly greater target-word gain for combined therapy compared to story-reading alone (*p* < 0.05, *f*^2^ = 0.17, medium effect) 5. Syntactic recognition test Significantly greater target-word gain for combined therapy compared to story-reading alone (*p* < 0.05, *f*^2^ = 0.20, medium effect) No significant order effects	Not measured	Medium quality
Storkel et al. (2019), US	Randomized alternating treatment crossover design with 2–3 weeks in between to measure learning retention	21 males & 13 females diagnosed with DLD as part of the study (age: 5; 0–6 ;2)	Story read to child + Explicit semantic approach	Dosage to achieve 36 target-word exposures varied by no. of therapy sessions per word (4, 6, 9) & word exposures per session (9, 6, 4). Total: 160–299 min 60 target words, 10 per approach, multiple presentations	**Provider**: University SLT & SLT students **Mode**: Face-to-face individual sessions in English **Location**: Mostly room in mainstream school	Within and between group, pre-post comparison using a researcher-created target-word definition test to assess expressive vocabulary	Within group Significant target-word gain from all dosage-delivery variations: •Variation 1: *p* = 0.002 •Variation 2: *p* < 0.0001 Between group No significant difference in scores between dosage delivery variations Effect sizes not reported	Approx. 60% loss in gains at 1-week follow up rising to 70% at 3-week follow up	High quality

^*^Based on JBI quality appraisal rating (see Appendix A).

LI, Language Impairment; SLI, Specific Language Impairment; DLD, Developmental Language Disorder.

**Table 4:** Summary of studies included in review – orthography-based vocabulary intervention approach.

**Study**	**Participants**	**Intervention**	**Design**	**Dosage**	**Delivery**	**Outcome measures**	**Results**	**Follow-up**	JBI quality rating^*^
Best (2005), UK	2 males and 3 females, with pre-identified mixed profiles of language/learning needs (age 6;10- 10;7)	Child introduced to digital aid to independently sound out initial letter of target words + Regular specialized language/learning input (specifics unclear)	Pre-post comparison	6 × 60 min sessions once a week for 6 weeks Total duration: 360 min/6 h 27–30 target words, multiple presentations	**Provider**: University SLT **Mode**: Combined digital & face-to-face individual sessions. Regular updates to child's SLT & teaching staff **Location**: Setting unknown	1. Individual pre-post comparison using a researcher-created picture-naming test of target words to assess expressive vocabulary 2. Individual pre-post comparison using the standardized Word Finding Vocabulary Test (Renfrew, 1995) to test expressive vocabulary of single words and Test of Word Finding in Discourse (German, 1991) to assess expressive vocabulary in sentences 3. Individual pre-post comparison using the standardized British Picture Vocabulary Scales (BPVS, Dunn et al., 1982) to assess receptive vocabulary 4. Pre-post comparison of group average percentage score for literacy (reading) & numeracy using child, parent, teacher & therapist completed visual scale “views questionnaire”	1. Picture naming •Significant gain for target words (*p* < 0.025 for all children) •No significant changes for control words 2. Standardized test of expressive vocabulary. Significant gain for 2 out of 5 children 3. Standardized test of receptive vocabulary. No significant change 4. Literacy (reading) & numeracy. No significant change in numeracy score from pre-test (51%) to post-test (51%) or reading score from pre-test (56%) to post-test (63%) Effect sizes not reported	Loss in gains at 6-month follow up	Low quality

^*^Based on JBI quality appraisal rating (see Appendix A).

### 3.3 Quality appraisal

Full details of the appraisal scores are available in Appendix A, and the overall quality rating for each study is included in the summary of studies tables (Tables 1–4, Column 3). Two studies were appraised as low quality and were omitted from quantitative synthesis (Best, 2005; Wing, 1990). Factors limiting the quality of these two studies included lack of control groups, small sample sizes, and ambiguity regarding access to ongoing specialist language support in addition to the experimental intervention.

### 3.4 Study properties

#### 3.4.1 Study design and sample sizes

The review identified one pre-post comparison case-study (Marks and Stokes, 2010, *n* = 1), two case-series reporting on pre-post comparisons of multiple individual case-studies (Best, 2005, *n* = 5; Parsons et al., 2005, *n* = 2), and one pre-post comparison group study (Ardanouy et al., 2023, *n* = 8). All the case-studies used control words as comparators in place of control groups.

Five controlled group studies were identified (Best et al., 2018, *n* = 11; Wright, 1993, *n* = 4; Motsch and Marks, 2015, *n* = 78; Nash and Donaldson, 2005, *n* = 16; Wing, 1990, *n* = 10). Two of the controlled studies were randomly assigned (Best et al., 2018; Motsch and Marks, 2015), while the remainder were matched by language profiles with the justification that population heterogeneity meant there was difficulty recruiting sufficient children with comparable characteristics.

Seven studies adopted a randomized crossover design to mitigate population heterogeneity. Interventions were delivered at different time periods, and three of the studies included a no-intervention period between interventions (Break between interventions: Best et al., 2021, *n* = 20; Steele et al., 2013, *n* = 12; Storkel et al., 2019, *n* = 34; No break: Lowman and Dressler, 2016, *n* = 12; Smeets et al., 2014, Study 1 *n* = 28 & Study 2 *n* = 21; Zens, 2009, *n* = 19). One of the crossover studies analyzed both individual and group effects (Best et al., 2021).

A pattern of chronology emerged in that over half of the studies (11) built on work covered in earlier studies in the review, enabling preliminary findings to be replicated, extended or generalized to a larger sample.

#### 3.4.2 Participant information

Half of the studies (8) identified participants as presenting with an existing diagnosis of Specific Language Impairment (SLI). The criteria for SLI as defined by Stark and Tallal (1981) is based on a discrepancy profile, with language ability significantly below the child's cognitive or chronological age; non-verbal ability within the normal range on standard scores; and typical hearing, sensory and socioemotional development. Whilst participants appeared to align to these diagnostic criteria for SLI within the studies reviewed, wide variation in assessments used and thresholds for diagnosis were noted. Although this weakens the strength of the synthesis, this diagnostic variability for SLI is typical both academically and clinically (Aram et al., 1993).

Three studies described their samples as children with an existing diagnosis of Language Impairment (Marks and Stokes, 2010; Steele et al., 2013; Wing, 1990). The criteria matched that of SLI as described in the above paragraph.

Four studies referred to their sample as presenting with DLD (Ardanouy et al., 2023; Best et al., 2018, 2021; Storkel et al., 2019). DLD deploys more descriptive diagnostic criteria than SLI, focusing on the sustained social and educational impact of pervasive language difficulties and allowing for certain co-morbidities (Bishop et al., 2017). Ardanouy et al. (2023) recruited children with an existing DLD diagnosis whilst the others diagnosed as part of their studies. Overall, the characteristics of participants aligned with that of SLI, apart from one child in the study by Ardanouy et al. (2023) who also presented with attention deficit hyperactivity disorder (ADHD).

One study defined their sample using descriptive profiles of existing language needs (Best, 2005). Characteristics aligned with that of SLI, apart from one child who also presented with ADHD and moderate learning difficulties.

All children in the reviewed studies were identified by investigators as presenting with vocabulary difficulties. The assessments used, the threshold for identification, and the nature of the difficulty (e.g., receptive and/or expressive) varied widely between the studies.

Only two studies provided socio-economic information. Steele et al. (2013) reported that most participants were African American (96%) and received subsidized lunches (85%). Parent education was also reported by Steele et al. (2013) with parents typically having a college degree, partial college accreditation, or high school diploma. Storkel et al. (2019) referred to most children in their study as being White, non-Hispanic (79%), and with married parents (53%). They also reported on parental education with most parents holding a college degree, partial college accreditation, or high school diploma.

Where reported, children across the studies were identified as monolingual, with the exception of one child studied by Best (2005) who spoke English and Spanish (replicating parents) and presented with difficulties in both languages. This child received intervention in English only.

#### 3.4.3 Target words

Lowman and Dressler (2016) used an established tiered framework (Beck et al., 2002) to select words that were described as unlikely to appear in everyday conversation but that would benefit from teaching as could be used across multiple contexts. Selection consensus was reached between the investigating SLT, SLT student and school SLT. Storkel et al. (2019) used words from a previously published study (Justice et al., 2005) which had adopted a similar target-word selection method as Lowman and Dressler (2016). Parsons et al. (2005) selected words from the UK national school curriculum.

For six studies, the target words were those considered by the research team to be relevant and meaningful (Ardanouy et al., 2023; Smeets et al., 2014: Study 1 & 2; Motsch and Marks, 2015; Wing, 1990; Zens et al., 2009). One of these six studies (Ardanouy et al., 2023) analyzed intervention effect according to target word categories (sports, animal, vegetable, school). Whilst another, Zens et al. (2009), ensured balance of word properties: meaning (category) and sound (syllable length, phonotactic probability).

For the remaining seven studies, target words were selected from pre-published vocabulary lists standardized for factors such as word properties, age of acquisition, frequency of occurrence, and familiarity. Of these seven, three studies (Best, 2005; Best et al., 2018, 2021) also targeted words of personal relevancy to the children, but the type and number varied for each child and outcomes were not included in their statistical analysis.

#### 3.4.4 Outcome measures

For ease of reading, Table 5 provides a summary of the measures used. Most studies (13) used researcher-created expressive target-word tests such as picture naming and word definitions to measure outcomes. Other vocabulary measures included researcher-created tests to assess receptive knowledge of target words and standardized expressive and/or receptive vocabulary tests.

**Table 5:** Summary of outcome measures.

	**Standardized**	**Non-standardized**
Expressive vocabulary	Best, 2005; Motsch and Marks, 2015; Parsons et al., 2005; Wing, 1990	Ardanouy et al., 2023; Best, 2005; Best et al., 2018, 2021; Lowman and Dressler, 2016; Marks and Stokes, 2010; Nash and Donaldson, 2005; Smeets et al., 2014 (Study 1 & 2); Steele et al., 2013; Storkel et al., 2019; Wright, 1993; Zens et al., 2009
Receptive vocabulary	Best, 2005; Parsons et al., 2005	Lowman and Dressler, 2016; Marks and Stokes, 2010; Nash and Donaldson, 2005; Parsons et al., 2005
Phonological awareness		Zens et al., 2009
Syntax	Motsch and Marks, 2015	Lowman and Dressler, 2016
Literacy & numeracy		Best, 2005

Generally, studies focused on vocabulary outcomes only, however, some did extend to standardized and non-standardized measures of phonological awareness (Zens et al., 2009), syntax (Lowman and Dressler, 2016; Motsch and Marks, 2015), literacy, and numeracy (Best, 2005). The range of study designs and small data sets meant it was not appropriate to synthesize or make conclusions around intervention effects beyond vocabulary outcomes.

#### 3.4.5 Follow up (maintenance of outcomes effects)

Of the six studies that measured changes in vocabulary scores from post-therapy to follow-up, five reported loss (Best, 2005: at 6 months follow-up; Best et al., 2021: at 6 weeks; Motsch and Marks, 2015, at 4 months; Storkel et al., 2019: at 1 week and 2 weeks; Wright, 1993: at 1 month) and one reported either no change or loss depending on time measured (Ardanouy et al., 2023: vegetable vocabulary–no change at 1.5 months; animals–no change at 3 months; sports–loss at 4.5 months).

No studies reported losses that marked a return to pre-therapy scores.

### 3.5 Intervention characteristics

#### 3.5.1 Intervention type

Studies in this review were grouped into four intervention approach types: phonological and/or semantic interventions (8 studies), story-based interventions (3 studies), story-based with semantic and/or phonological interventions (4 studies), orthography-based interventions (1 study).

Studies exploring phonological and semantic approaches focused on both teaching the child phonological and semantic word features and cuing the child to self-generate information about word sounds and meaning. Wing (1990) compared phonological and semantic approaches, whereas Wright (1993), Motsch and Marks (2015), and Parsons et al. (2005) investigated combined strategies. Zens et al. (2009) studied the influence of ordering phonological and semantic approaches. Best et al. (2018) utilized word webs, which are visual diagrams to cue, map, and record phonological and semantic word features. Best et al. (2021) also used word webs but considered differential responses to intervention based on presenting profiles. Ardanouy et al. (2023) added contextualization to phonological and semantic word-learning strategies by connecting to personal experiences and considering words in multiple environments with a range of communication partners. All the studies in this category used objects and/or pictures to support word learning, and all bar Wing (1990) and Wright (1993) also used the written form of the target word.

The second intervention type involved exposure to stories to provide contextual cues for vocabulary learning. Nash and Donaldson (2005) compared incidental word learning through exposure to pre-recorded narrated stories and corresponding picture books against explicit exposure to semantic word features. Smeets et al. (2014) conducted two studies: one comparing the child being exposed to narrated electronic story books with either static images or videos with background and music audio, and the other comparing static picture vs. video narrated electronic written story books either with or without background noise and music.

The third intervention approach combined stories to contextualize target words with explicit exposure to phonological and/or semantic word features. In Marks and Stokes (2010) an adult introduced a spoken story with corresponding pictures, followed by explicit teaching of semantic features and supporting of story generation. In Steele et al. (2013), children were supported by an adult to read story text containing the target word as well as learning and generating specific phonological and/or semantic word features. Lowman and Dressler (2016) exposed children to pre-recorded phonological, semantic, and syntactic word features via an iPod, in addition to the children independently reading storybooks containing the target words, versus independent storybook reading alone. Storkel et al. (2019) introduced target words through shared adult-child reading of written story picture books in addition to explicitly teaching semantic word features.

The final intervention reviewed used alphabetic letters to support vocabulary skills. Best (2005) introduced a digital aid that children could use to sound out the initial letters of target words presented in picture form, SLT support was provided as required.

Four studies (Best, 2005; Lowman and Dressler, 2016; Motsch and Marks, 2015; Zens et al., 2009) mentioned the continuation of ongoing specialist language support from the children's SLT and/or school in addition to the experimental interventions. Details were brief making the influence of the additional support difficult to ascertain, with the exception of Motsch and Marks (2015) where only a sub-sample received additional support and where intervention effects were maintained even once data for the additional-support cohort was removed. Other studies explicitly stated that no other specialist support was accessed (Best et al., 2018, 2021; Ardanouy et al., 2023), whilst the remaining studies did not comment either way.

#### 3.5.2 Intervention dosage

Interventions ranged from 40 min spread over 2 days (Nash and Donaldson, 2005) to 900 min/15 h spread over 5 months (Motsch and Marks, 2015).

Most studies (9) delivered intervention weekly or biweekly lasting 30–60 min over 4–6 weeks. Three studies provided interventions three or more times a week (Wright, 1993; Parsons et al., 2005; Wing, 1990) lasting 20–60 min over 3–10 weeks. Three studies provided insufficient detail to calculate intervention duration (Smeets et al., 2014: Study 1 & 2; Steele et al., 2013).

One study (Storkel et al., 2019), building on previous work that identified 36 exposures per target word as the optimal for vocabulary interventions (Storkel et al., 2017), compared intervention frequencies for optimal exposure. Dosage delivery ranged between 10 and 23 sessions lasting 13–16 min over 5–12 weeks; no differential effect of dosage on intervention outcomes was found.

The number of target words varied between studies ranging from 4 to 90 words per child per intervention. There was a general correlation between intervention dosage and number of words targeted. However, as number of exposures to target words was inconsistently reported across the studies, findings could not be pooled.

#### 3.5.3 Intervention provider

In most studies (9), intervention was provided by university-affiliated SLTs who were the primary investigators (ranging from professors to doctoral students).

The exceptions were four studies where the SLT was affiliated to the child's school or health provider (Marks and Stokes, 2010; Parsons et al., 2005; Wing, 1990; Wright, 1993), one study where the affiliation of the SLTs delivering the intervention was unclear (Motsch and Marks, 2015), and two studies where intervention was delivered by psychology students who were the primary investigators (Masters and PhD: Smeets et al., 2014).

In three studies the SLT was supported in delivering the intervention by SLT students (Lowman and Dressler, 2016; Steele et al., 2013; Storkel et al., 2019) and in one study by educational psychologists (Ardanouy et al., 2023).

Two studies mentioned that the intervention details were shared with caregivers and teaching staff for follow-up (Motsch and Marks, 2015; Parsons et al., 2005) and one mentioned follow-up by the child's SLT, caregivers and teaching staff (Best, 2005); none of these studies quantified the follow-up support which limits replicability.

#### 3.5.4 Intervention mode

In most studies (9), intervention was delivered as face-to-face individual sessions with the intervention provider. Four further studies (Ardanouy et al., 2023; Wing, 1990; Wright, 1993; Zens et al., 2009) delivered intervention through face-to-face group sessions. One study (Motsch and Marks, 2015) compared face-to-face individual sessions and face-to-face group sessions, and found greater expressive vocabulary gain with group therapy and greater syntax gain (expressive and receptive) with individual therapy.

Three studies used multimedia interventions (Smeets et al., 2014: Study 1 & 2–television; Lowman and Dressler, 2016–iPod; Best, 2005–digital aid sounding out alphabet letters). In Lowman and Dressler (2016), children viewed videos containing text, pictures and animation related to target words in addition to reading storybooks embedded with target words, and reported greater gains than with reading alone. Smeets et al. (2014) investigated the differential effect of multimedia by comparing word learning using narrated e-books with static pictures versus narrated e-books with animation, both conditions were presented either with or without background music/sound. Although no mode effect was found at the group level, a significant correlation emerged between increased language severity and negative influence of background sounds. Finally, Best (2005) adopted a hybrid model with the digital aid introduced during face-to-face individual sessions with a SLT, and found greater gains with the aid than without.

#### 3.5.5 Intervention location

One study was conducted in a specialist clinic (Ardanouy et al., 2023), five studies were conducted in a quiet room in a specialist school (Motsch and Marks, 2015; Smeets et al., 2014: Studies 1 & 2; Wing, 1990; Wright, 1993), with the remaining studies undertaken in mainstream schools, bar one study which did not confirm location (Best, 2005).

### 3.6 Quantitative synthesis of intervention outcomes

Meta-analysis was precluded by variations in the collection, analysis and reporting of quantitative data compounded by the heterogeneity of study design, clinical populations, diagnostic criteria, and intervention characteristics. Instead, outcomes were summarized according to the Synthesis Without Meta-analysis (SwiM) guidelines (Campbell et al., 2020) with *p*-value and effect size ranges reported.

Across the review, intervention type was the only intervention characteristic consistently identified as a study variable, and vocabulary gain was the only consistently reported intervention effect measure. As a result, quantitative findings have been synthesized and compared according to the effect of intervention type on vocabulary outcomes. Table 6 provides a summary of this quantitative synthesis with further detail.

**Table 6:** Summary of quantitative synthesis.

		**Intervention**		
		**Phonological (P) and/or Semantic (S) - Eight studies**	**Story - Three studies**	**Story** **+** **P and/or S - Three studies**
	Expressive vocabulary	**Phonological and Semantic combined (Five studies)**Significance range: *p* < 0.0001 to 0.01*Source:* Ardanouy et al., 2023 (n = 8); Best et al., 2018 (n = 11); Wright, 1993 (n = 4); *Motsch and Marks, 2015 (n = 78); Zens et al., 2009 (n = 19)*Effect size: Large*Source:* Ardanouy et al., 2023; Motsch and Marks, 2015; Zens et al., 2009	**Story (Two studies)** Significance: *p* < 0.001–0.05 *Source: Nash and Donaldson, 2005 (n = 16); Smeets et al. (2014) Study 1 (n = 28)* Effect size: Large. *Source: Smeets et al., 2014, Study 1 (n = 28)*	**Story** **+** **P&S (Two studies)**Significance range: *p* = 0.028–0.05*Source: Lowman and Dressler, 2016; (n = 18); Steele et al., 2013 (n = 12)*Effect size: Small*Source: Lowman and Dressler, 2016*
Outcome			**Semantic only (Three studies)**Significance range: *p* = 0.004 to < 0.01*Source: Best et al., 2021 (n = 20); Nash and Donaldson, 2005 (n = 16); Zens et al., 2009 (n = 19)*Effect size range: Medium to large*Source: Best et al., 2021; Zens et al., 2009*		**Story** **+** **S (Two studies)**Significance range: *p* = < 0.0001 to 0.002*Source: Steele et al., 2013 (n = 12); Storkel et al., 2019 (n = 34)*Effect size not reported.
				**Phonological only (One study)**Significance: *p* = 0.001Effect size range: Large.*Source: Zens et al., 2009 (n = 19)*		
			Receptive vocabulary	**Phonological and Semantic combined (One study)**Significance range: *p* < 0.001 to < 0.01*Source*: *Parsons et al., 2005 (n = 2)*Effect size not reported	**Story (One study)** Significance range: *p* < 0.001 to < 0.05 (Spoken word recognition & Meaning recognition) *Source: Nash and Donaldson, 2005 (n = 16)* Effect size not reported	**Story** **+** **P&S (One studies)**Significance: *p* < 0.01*Source: Lowman and Dressler, 2016 (n = 12)*Effect size range: Small to large*Source: Lowman and Dressler, 2016 (n = 12)*
					**Semantic only (One study)**Significance: *p* < 0.01*Source: Nash and Donaldson, 2005 (n = 16)* Effect size not reported		

#### 3.6.1 Phonological and/or semantic interventions (8 studies: 3 high design quality, 5 medium)

Six studies found significant vocabulary gain when phonological and semantic interventions were combined. Of these, three studies showed gain on expressive target-word scores (Ardanouy et al., 2023; Best et al., 2018; Wright, 1993), one showed gain on both target-word and non-target word tests of expressive vocabulary Zens et al., 2009, one showed gain on standardized expressive vocabulary tests (Motsch and Marks, 2015) and one showed gain on receptive target-words but not on standardized tests of receptive or expressive vocabulary (Parsons et al., 2005). With semantic intervention alone, three studies found significant gain on expressive vocabulary (target-word scores: Best et al., 2021; Nash and Donaldson, 2005, target and non-target word scores: Zens et al., 2009). With phonological intervention alone, one study found significant gain on expressive vocabulary (target and non-target word scores: Zens et al., 2009).

Two studies evaluated the effects of ordering phonological and semantic interventions (Best et al., 2021; Zens et al., 2009). Neither found an intervention order effect. However, Best et al. (2021) reported greater target-word gain during the semantic than the phonological intervention phase at the group level. In contrast, Zens (2009), found significantly greater target-word naming gain after day one with the phonological intervention but not with the semantic intervention (though the significance was not maintained over the course of the phonological intervention).

Best et al. (2021) also explored the extent to which individual children's responses to semantic and/or phonological intervention were influenced by their profile of need at baseline. Ninety-one percent of children (10/11) with semantic and phonological needs showed expressive target-word gain from either phonological, semantic, or combined intervention; two-thirds of the children (4/6) with relatively more semantic difficulties benefited from semantic but not phonological intervention and two-thirds with relatively more phonological difficulties benefited from phonological but not semantic intervention (2/3). Altogether, Best et al. (2021) identified that 90% (18/20) of children in their study showed expressive target-word gain after accessing the semantic and/or phonological components of word web intervention.

#### 3.6.2 Story-based interventions (3 studies: all with medium design quality)

One study (Nash and Donaldson, 2005) investigated incidental word learning through exposure to narrated picture stories and reported significant target-word gain on expressive and receptive vocabulary measures. When compared to an explicit semantic intervention, greater gain was reported from the semantic intervention on both expressive and receptive target-word measures.

Two studies explored the effect of exposure to narrated electronic story books on target words. One reported greater gain for targeted words introduced as a picture or animated e-story compared to control words (Smeets et al., 2014, Study 1), and the other found a significant correlation between increased language severity and the negative influence of background sound in e-books (Smeets et al., 2014, Study 2).

#### 3.6.3 Stories + phonological and/or semantic interventions (3 studies: 1 high quality, 2 medium)

Two studies reported significant gains on expressive and receptive target-word measures when stories were combined with phonological and semantic interventions: in one study (Steele et al., 2013) therapy was provided in person, while in the other (Lowman and Dressler, 2016) multimedia was used. Two studies found significant gain on expressive measures when stories were combined with semantic interventions (Steele et al., 2013; Storkel et al., 2019). It is worth noting that Storkel et al. (2019) reported a significant correlation between higher language scores and greater word-learning gain.

## 4 Discussion

This review considered available studies targeting vocabulary skills in children with DLD in order to identify and describe key intervention features. What follows is a discussion of the characteristics of the vocabulary interventions that have been reviewed and the effects on word learning, consideration is also given to the design of the studies that generated the reported findings.

### 4.1 The benefits of an integrated therapeutic model

The use of combined phonological and semantic word-learning strategies was the most frequently evaluated intervention, with most of the reviewed studies having a high-quality design and demonstrating statistically significant expressive vocabulary gain with a large effect size. Studies investigating semantic or phonological approaches alone also demonstrated gain but less consistently. These findings align with the connectionist model of language processing and the proposal that vocabulary difficulties in children with DLD may stem from both the processing of sound and meaning, and the interactions between them (Chiat, 2001).

There were some indications of the benefit of explicit sound-letter cueing to support phonological processing during word learning, however, as findings were restricted to one study which was appraised as having a low design quality, more research is needed for firm conclusions to be drawn.

There were stronger indications that integrating contextual story narratives with explicit phonological and semantic cues strengthened vocabulary gains, as demonstrated in four studies of medium to high quality. This is in keeping with the cross-situational model of language acquisition which proposes that children's vocabulary is enhanced when information is presented in multiple contexts because patterns of co-occurrence strengthen word learning (Roembke et al., 2023). However, exposure to stories alone was not as effective as explicit cuing in improving word-learning. This indicates that the interactive, conversational aspect of narratives may be key in supporting children with DLD, as highlighted in the work by Law et al. (2022) who explored the influence of social context for children with DLD.

Taken together, these results align with current thinking around the benefits of integrating intervention approaches to improve outcomes for children with DLD. For example, Baron and Arbel (2022a) discuss how differential learning systems are engaged when explicit and implicit intervention approaches are combined. According to Baron and Arbel (2022a), explicit learning, where the child is systematically taught facts, can address underlying processing difficulties in children with DLD. However, there is a risk of dependency on rote-learning and memorisation which incidental learning can mitigate through inferencing and generalization opportunities. Applying this theory to word learning, by combining explicit phonological-semantic vocabulary strategies with social narratives, children can use explicit and implicit cues to acquire, apply, and refine sound-meaning associations.

Effective integration of explicit and incidental word-learning strategies is not limited to exposing DLD children to targeted words within narratives during therapy. Research into word recall has demonstrated that word retention in children with DLD is influenced not only by the robustness of the initial learning phase but also meaningful retrieval and practice opportunities (Kueser et al., 2021; Leonard et al., 2020). It is important to consider that most studies in this review that reported on maintenance of word-learning gains observed depreciation at follow-up relative to the gains made from pre- to post-intervention. This was regardless of the intervention approach used, indicating that there are other factors at play. One such factor could be the selection of target words as most studies used pre-set word lists, however, words from the child's personal life, interests, or school curriculum would have provided more incidental reinforcement opportunities. Studies that utilized curriculum and personal interest words did not include follow-up measures; therefore, implications for retention require further exploration.

The need to target functional words for meaningful and sustained word learning is further reinforced by indications in this review that on the whole therapy gains did not generalize beyond the directly targeted words. This was demonstrated by the minimal transfer of gains to control words or standardized vocabulary assessments.

Another strategy for integrating explicit and implicit word-learning opportunities that was evident in the reviewed interventions was the use of self-cuing. Most studies embedded self-cueing strategies where children were encouraged to independently apply word-learning techniques once they had been modeled and practiced with an adult. However, there was only one example where this strategy continued to be studied post-therapy and even then, it was informally rather than through standardized observations and evaluation (Best et al., 2021). Given that autonomous self-cueing has been identified as an area of implicit learning that is compromised in children with DLD due to poor executive functioning and language planning (Baron and Arbel, 2022b; Senter, 2022), further research is warranted.

Combining individual and group therapy sessions can also provide children with DLD the opportunity to benefit from explicit and implicit word-learning opportunities. Most studies in this review adopted either individual or group therapy, however, one study directly compared the two approaches and found greater vocabulary gain at word level with group therapy and at sentence level with individual therapy (Motsch and Marks, 2015). This differential response aligns with findings for language therapy more generally, and the proposed activation of separate learning mechanisms. Group therapy is considered to have the advantage of targeting words in peer discourse with multiple communication partners, whereas individual therapy enables more tailored support (Watt and White, 2018). Further exploration of the effects of individual versus group vocabulary therapies is required.

### 4.2 Individualized approaches to provide intervention that aligns with needs

A key finding that emerged from the reviewed vocabulary intervention studies was the importance of considering children's presenting profiles. This was most evident in the study by Best et al. (2021) which completed both group- and individual-level analyses. As a group, children with DLD showed significantly greater word-learning gain from explicit semantic interventions compared with explicit phonological therapy. However, case-series analysis indicated differential patterns of response depending on presentation. Children presenting with both phonological and semantic difficulties responded to both or either intervention. However, children presenting with greater semantic than phonological difficulties tended to benefit from semantic but not phonological therapy. The converse was also true with phonologically-impaired children tending to respond to phonological but not semantic therapy. Crucially, some children did not respond to either intervention, indicating that word-learning strategies for children with DLD cannot be chosen solely on presenting profiles.

Threshold of intervention gain is another feature that may have differential implications depending on the language profile of a child with DLD. One study (Storkel et al., 2019), identified that children with more severe language difficulties were less responsive to combined explicit and implicit word-learning strategies. This built on previous work by Storkel et al. (2017) exploring dosage, which identified a plateau in word-learning gains in children with DLD in response to increased intervention exposure. The ceiling appeared related to language proficiency, with children who had more severe needs reaching ceiling of benefit sooner. Taken together, this indicates the need for an individualized approach for vocabulary interventions and careful monitoring of progress when considering the optimal dosage and level of response.

Whilst the use of multimedia during word learning was found to be of benefit for children with DLD (Best, 2005; Lowman and Dressler, 2016; Smeets et al., 2014), this was another area where the nature of responses differed according to the profile of language needs. More specifically, Smeets et al. (2014) demonstrated that children with more severe language difficulties benefited from digital images and videos to support word-learning only when extraneous background music and sounds were removed. Given that remote working measures introduced during COVID-19 have led to a subsequent rise in digital practice to support children with DLD (McCartney and Forbes, 2023; Ansari et al., 2022), further research is needed to determine how technology may support individual needs.

## 5 Limitations

### 5.1 Limitation of reviewed intervention studies

Intervention quality was generally found lacking in the reviewed interventions. Of the 16 studies reviewed, only four were appraised as being of high quality; methodological weaknesses included small sample sizes, weak or lack of control measures, and omission of effect size calculations. It is promising that the later-dated studies were the ones of high quality (2015 onwards), and worth noting that they generally built on work from the earlier more exploratory studies indicating the value of the earlier work. A key point, however, is that one of the high-quality RCTs compared intervention effect at the group level with the individual level and reported discrepancies (Best et al., 2021). This is inevitable given the heterogeneity of the DLD population; however, as advised by numerous researchers in the field, it can be addressed by adopting multi-method case-series and controlled group trial approaches. This would maintain research rigor whilst providing findings that can be applied to a heterogeneous population as is required by SLTs delivering interventions in practice (Best et al., 2019; Forsythe et al., 2022).

The heterogeneity of the population was further compounded between studies by differences in diagnostic terminology. The use of Language Impairment or Specific Language Impairment as diagnostic labels was evident in most studies published before 2018, whereas all studies from 2018 and after adopted DLD. This mirrors a broader diagnostic shift by clinicians and academics away from identifying language impairment based on discrepancies between language and intelligence scores (Stark and Tallal, 1981; Tomblin et al., 1997) to a more descriptive approach focusing on the sustained social and educational impact of language difficulties (Bishop et al., 2017). While the rationale is well-documented (Royal College of Speech Language Therapists: RCSLT, 2021; McGregor et al., 2020), it introduces further heterogeneity among already varied study samples, which in turn impacts the comparability and synthesis of findings.

Poor consideration of the impact of the intervention provider and setting was a further limitation of the reviewed studies. Only three studies extended intervention to the home and school environment (Best, 2005; Motsch and Marks, 2015; Parsons et al., 2005), and none quantified this additional support. Wider research involving children with a range of communication needs has demonstrated the benefits of vocabulary learning when intervention strategies are embedded within the home and classroom (Throneburg et al., 2000; Ebbels et al., 2019; Roberts and Kaiser, 2011). Theoretically, this aligns with the cross-situational model of language acquisition (Roembke et al., 2023), which emphasizes the value to children of repeated vocabulary exposure across physical and social environments. Given the shortage of SLTs to support children with DLD (Christopulos and Redmond, 2023; Gibbons, 2021), and the findings from this review that children experience deterioration in word-learning gains once intervention is withdrawn, there is a need for research that robustly explores long-term home-school reinforcement of vocabulary interventions.

Another limitation across most of the reviewed studies was the poor reporting of children's socioeconomic status. This has implications for research and practice given that low SES is considered a risk factor for DLD and may influence access to and response to treatment (Bishop et al., 2017; McGregor, 2020).

### 5.2 Limitations of systematic review

Variation in study design meant the Synthesis Without Meta-analysis (SWiM, Campbell et al., 2020) guidelines were applied and quantitative synthesis was limited to reporting *p*-value and effect size range. Unlike meta-analysis, this approach is limited in analyzing the extent of variation in *p*-values and effect sizes among studies or revealing causes of heterogeneity. However, it provides greater insight into the measurable impact of an intervention on outcomes and influential factors than a qualitative summary alone (Siedler, 2022).

To assist with the synthesis of the findings, this review was limited to studies reported in English only. It is acknowledged that this excludes a body of work that may be relevant.

## 6 Recommendations

### 6.1 Research implications

Based on the systematic review findings, the following recommendations are made for future research on vocabulary interventions for children with DLD:

1. Combine case-study and controlled group methods to generating findings relevant to a heterogeneous population whilst maintaining research rigor.
2. Use controlled variable studies to explore the impact of explicit sound-meaning word-learning strategies reinforced implicitly over time across providers (teachers, caregivers) and settings (school, home).
3. Select target words that are of relevance for children with DLD and that they are likely to be re-exposed to outside of the therapy environment.
4. Recruit more representative samples with diverse socioeconomic status and ethnicity, with detailed reporting of participant characteristics.

### 6.2 Clinical implications

The following recommendations from this systematic review are relevant for professionals supporting children with DLD:

1. As word-learning gains are typically restricted to directly targeted words, practitioners, educators, and caregivers should work in partnership with children who have DLD to identify the most relevant target words. These should relate to the children's interests, curriculum, and routines, so that the words are useful across multiple environments and with various communication partners.
2. Children with DLD may show greater response to the phonological, semantic and phonological-semantic cues that match the areas they struggle with. However, most children will respond to phonological-semantic cues combined, and this should form the start of therapy which can then be refined depending on how the child responds. Both Best et al. (2018) and Parsons et al. (2005) provide detailed protocols for phonological-semantic interventions (see also Branagan and Parsons, 2021, Word Aware 3).
3. For maximum benefit, explicit phonological-semantic word-learning strategies should be targeted both individually and in groups and then reinforced by providing frequent opportunities for generalization. Generalization strategies should include exposure to words in conversational narratives, encouragement of self-cuing, and on-going opportunities to hear and use the words at home and school.
4. When planning interventions, take into consideration that children with DLD require repeated target-word exposures during combined explicit and implicit word-learning to make significant vocabulary gains. The maximum beneficial exposure will vary between children depending on language severity, therefore, joint SLT-school-home planning for intervention delivery and monitoring is important.
5. The use of multimedia to support vocabulary skills, i.e., audio, images, video, is advocated. However, multimedia word-learning should be planned carefully and monitored closely, as each child is likely to respond differently depending on their language and learning profiles, as well as individual preferences.

## 7 Conclusions

This review considered the available research on vocabulary interventions to understand the intervention features leading to vocabulary gain in children with DLD. The range and quality of studies are currently restricted but enabled some conclusions to be drawn.

The results suggest word-learning benefits from integrating explicit phonological-semantic prompts with implicit contextual cues. Factors that may influence outcome include the child's language and learning profile, vocabulary relevance, reinforcement opportunities, use of self-cuing, and peer-modeling.

More research is needed across all aspects of vocabulary interventions for children with DLD, with gaps most evident in relation to the influence of socioeconomic status and generalization to the home and school environment.

This paper is important for professionals working with children who have DLD, as it offers practical, evidence-based guidance for applying research to meet children's individual word-learning needs. The findings also have bearing for researchers and academics as they guide the direction of future research.

## Data Availability

The original contributions presented in the study are included in the article/Supplementary material, further inquiries can be directed to the corresponding author.

## References

[B1] Alduais A. Majorano M. Andrés-Roqueta C. Hamaguchi P. Persici V. Qasem F. (2022). Conceptualizing, defining, and assessing pragmatic language impairment in clinical settings: a scoping review. Infant Child Dev. 31:e2368. 10.1002/icd.2368

[B2] Anglin J. M. Miller G. A. Wakefield P. C. (1993). Vocabulary development: a morphological analysis. Monograp. Soc. Res. Child Dev. 1993:186. 10.2307/1166112

[B3] Ansari R. Loraine E. Gréaux M. (2022). Impact of COVID-19 on digital practice in UK paediatric speech and language therapy and implications for the future: a national survey. Int. J. Lang. Commun. Disord. 57, 1112–1129. 10.1111/1460-6984.12750 35925005 PMC9538739

[B4] Aram D. M. Morris R. Hall N. E. (1993). Clinical and research congruence in identifying children with specific language impairment. J. Speech Lang. Hear. Res. 36, 580–591. 10.1044/jshr.3603.580 8331914

[B5] Ardanouy E. Delage H. Zesiger P. (2023). Effectiveness of a group intervention for lexical enrichment in 6-to-10-year-old children with developmental language disorder. Child Lang. Teach. Ther. 39, 218–233. 10.1177/02656590231188523

[B6] Baron L. S. Arbel Y. (2022a). An implicit–explicit framework for intervention methods in developmental language disorder. Am. J. Speech-Lang. Pathol. 31, 1557–1573. 10.1044/2022_AJSLP-21-00172 35446629 PMC9531931

[B7] Baron L. S. Arbel Y. (2022b). Inner speech and executive function in children with developmental language disorder: implications for assessment and intervention. Perspect. ASHA SIG 7, 1645–1659. 10.1044/2022_PERSP-22-00042 38957614 PMC11218747

[B8] Beck I. L. McKeown M. G. Kucan L. (2002). Bringing Words to Life. New York, NY: Guilford Press.

[B9] Best W. (2005). Investigation of a new intervention for children with word-finding problems. Int. J. Lang. Commun. Disord. 40, 279–318. 10.1080/13682820410001734154 16195190

[B10] Best W. Fedor A. Thomas M. S. (2015). Intervening to alleviate word-finding difficulties in children: case series data and a computational modelling foundation. Cogn. Neuropsychol. 32, 133–168. 10.1080/02643294.2014.1003204 25711886

[B11] Best W. Hughes L. Masterson J. Thomas M. S. Howard D. Kapikian A. . (2021). Understanding differing outcomes from semantic and phonological interventions with children with word-finding difficulties: a group and case series study. Cortex 134, 145–161. 10.1016/j.cortex.2020.09.030 33279809

[B12] Best W. Hughes L. M. Masterson J. Thomas M. Fedor A. Roncoli S. . (2018). Intervention for children with word-finding difficulties: a parallel group randomised control trial. Int. J. Speech Lang. Pathol. 20, 708–719. 10.1080/17549507.2017.1348541 28756691

[B13] Best W. Ping Sze W. Edmundson A. Nickels L. (2019). What counts as evidence? Swimming against the tide: valuing both clinically informed experimentally controlled case series and RCTs in intervention research. Evid. Based Commun. Assess. Interv. 13, 107–135. 10.1080/17489539.2019.1597444

[B14] Biemiller A. Slonim N. (2001). Estimating root word vocabulary growth in normative & advantaged populations: evidence for a common sequence of vocabulary acquisition. J. Educ. Psychol. 93:498. 10.1037/0022-0663.93.3.498

[B15] Bishop D. V. Snowling M. J. Thompson P. A. Greenhalgh T. (2017). Phase 2 of CATALISE: a multinational and multidisciplinary Delphi consensus study of problems with language development: terminology. J. Child Psychol. Psychiatry 58, 1068–1080 10.1111/jcpp.12721 28369935 PMC5638113

[B16] Bleses D. Makransky G. Dale P. S. Højen A. Ari B. A. (2016). Early productive vocabulary predicts academic achievement 10 years later. Appl. Psycholinguist. 37, 1461–1476. 10.1017/S0142716416000060

[B17] Branagan A. Parsons S. (2021). Word Aware 3: Teaching Vocabulary in Small Groups for Ages 6 to 11. London: Routledge. 10.4324/9781003159377

[B18] Broedelet I. Boersma P. Rispens J. (2023). Implicit cross-situational word learning in children with & without DLD and its relation to lexical-semantic knowledge. Front. Commun. 8:1021654. 10.3389/fcomm.2023.1021654

[B19] Campbell M. McKenzie J. E. Sowden A. Katikireddi S. V. Brennan S. E. Ellis S. . (2020). Synthesis without meta-analysis (SWiM) in systematic reviews: reporting guideline. BMJ 368:l6890. 10.1136/bmj.l6890 31948937 PMC7190266

[B20] Chiat S. (2001). Mapping theories of developmental language impairment: premises, predictions and evidence. Lang. Cogn. Process. 16, 113–142. 10.1080/01690960042000012

[B21] Christopulos T. T. Redmond S. M. (2023). Factors impacting implementation of universal screening of developmental language disorder in public schools. Lang. Speech Hear. Serv. Sch. 54, 1080–1102. 10.1044/2023_LSHSS-22-00169 37459613

[B22] Cirrin F. M. Gillam R. B. (2008). Language intervention practices for school-age children with spoken Language Disorders: a systematic review. Lang. Speech Hear. Serv. Schools 39:1. 10.1044/0161-1461(2008/012) 18162642

[B23] Dennis J. Law J. Charlton J. (2017). Speech and language therapy interventions for children with primary speech and/or Language Disorders: (Protocol). Cochrane Database System. Rev. 2017, 1–21. 10.1002/14651858.CD012490 PMC8407295 12918003

[B24] Dunn L. M. Dunn L. M. Whetton C. Pintillie D. (1982). The British Picture Vocabulary Scale. NFER-Nelson: Windsor.

[B25] Ebbels S. H. McCartney E. Slonims V. Dockrell J. E. Norbury C. F. (2019). Evidence-based pathways to intervention for children with Language Disorders. Int. J. Lang. Commun. Disord. 54, 3–19. 10.1111/1460-6984.12387 29696726

[B26] Esser G. Wyschkon A. Ballaschk K. Hänsch S. (2010). Potsdam–Illinois Test für Psycholinguistische Fähigkeiten (P-ITPA) [Potsdam–Illinois test for psycholinguistic abilities, P-ITPA]. Göttingen: Hogrefe

[B27] Forsythe R. Murphy C. A. Tulip J. Law J. (2022). Why clinicians choose their language intervention approach: an international perspective on intervention for children with DLD. Folia Phoniatr. Logopaedica 73,537–551. 10.1159/000513242 33508820

[B28] Georgan W. C. Hogan T. P. (2019). The many terms used for DLD. DLD and Me. Available at: https://dldandme.org/terminology/ (accessed January 26 2022).

[B29] German D. J. (1986). Test of Word Finding. Austin, TX: Developmental Learning Materials.

[B30] German D. J. (1989). Test of Word Finding. Dallas, TX: DLM Teaching Resources.

[B31] German D. J. (1991). Test of Word Finding in Discourse. Leicester: Taskmaster.

[B32] Gibbons M. (2021). Development and delivery of an educational intervention that increased teachers' awareness, knowledge, and actions related to Developmental Language Disorder (DLD) (M.Sc. thesis). Institute of Technology, Sligo. Available at: https://research.thea.ie/handle/20.500.12065/4518 (accessed January 26, 2024).

[B33] Glück C. W. (2011). Wortschatz- und Wortfindungstest für Sechs- bis Zehnjährige (WWT 6–10) [Test of vocabulary size and word-finding abilities for children between six and ten, WWT 6–10]. München: Elsevier.

[B34] Green L. (2020). The specific language impairment/developmental Language Disorders forum: fostering a discussion of terminology. Perspect. ASHA Spec. Interest Groups 5, 3–5. 10.1044/2019_PERSP-19-00184

[B35] Hartley C. Bird L. A. Monaghan P. (2020). Comparing cross-situational word learning, retention, and generalisation in children with autism and typical development. Cognition 200:104265. 10.1016/j.cognition.2020.104265 32259659

[B36] Hoffmann T. Glasziou P. Boutron I. Milne R. Perera R. Moher D. . (2014). Better reporting of interventions: template for intervention description and replication (TIDieR) checklist and guide. BMJ 348:1687. 10.1136/bmj.g1687 24609605

[B37] Justice L. Cain K. Jiang H. Logan J. Jia R. Jiang H. . (2018). Modeling the nature of grammar and vocabulary trajectories from prekindergarten to third grade. J. Speech Lang. Hear. Res. 61, 910–923. 10.1044/2018_JSLHR-L-17-0090 29642241

[B38] Justice L. M. Meier J. Walpole S. (2005). Learning new words from storybooks: An efficacy study with at-risk kindergartners. Lang. Speech Hear. Serv. Sch. 36, 17–32. 10.1044/0161-1461(2005/003) 15801505

[B39] Justice L. M. Schmitt M. B. Murphy K. A. Pratt A. Biancone T. (2014). The ‘robustness' of vocabulary intervention in the public schools: targets and techniques employed in speech–language therapy. Int. J. Lang. Commun. Disord. 49, 288–303. 10.1111/1460-6984.12072 24299516

[B40] Khan K. S. Logan J. Justice L. M. Bowles R. P. Piasta S. B. (2021). The contribution of vocabulary, grammar, and phonological awareness across a continuum of narrative ability levels in young children. J. Speech Lang. Hear. Res. 64, 3489–3503. 10.1044/2021_JSLHR-20-00403 34351810 PMC9128800

[B41] Kueser J. B. Leonard L. B. Deevy P. Haebig E. Karpicke J. D. (2021). Word-learning trajectories influence long-term recall in children with developmental Language Disorder and typical development. J. Commun. Disord. 94:106160. 10.1016/j.jcomdis.2021.106160 34768092 PMC8715761

[B42] Law J. Reilly S. McKean C. editors. (2022). Language Development: Individual Differences in a Social Context. Cambridge: Cambridge University Press. 10.1017/9781108643719

[B43] Leonard L. B. Deevy P. Karpicke J. D. Christ S. L. Kueser J. B. (2020). After initial retrieval practice, more retrieval produces better retention than more study in the word learning of children with developmental language disorder. J. Speech Lang. Hear. Res. 63:2763. 10.1044/2020_JSLHR-20-00105 32692599 PMC7872731

[B44] Lindsay G. Strand S. (2016). Children with language impairment: prevalence, associated difficulties, and ethnic disproportionality in an English Population. Front. Educ. 1:2. 10.3389/feduc.2016.00002

[B45] Lowman J. J. Dressler E. V. (2016). Effects of explicit vocabulary videos delivered through iPods on students with language impairments. J. Spec. Educ. Technol. 31, 195–206. 10.1177/0162643416673914

[B46] Marante L. Hall-Mills S. (2024). Exploring speech-language pathologists' perception of and individualized education program goals for vocabulary intervention with school-age children with language disorders. Lang. Speech Hear. Serv. Sch. 55, 368–380. 10.1044/2023_LSHSS-23-00078 38295301

[B47] Marks I. Stokes S. F. (2010). Narrative-based intervention for word-finding difficulties: a case study. Int. J. Lang. Commun. Disord. 45, 586–599. 10.3109/13682820903277951 19857187

[B48] Matte-Landry A. Boivin M. Tanguay-Garneau L. Mimeau C. Brendgen M. Vitaro F. . (2020). Children with persistent versus transient early language delay: language, academic, and psychosocial outcomes in elementary school. J. Speech Lang. Hear. Res. 63, 3760–3774. 10.1044/2020_JSLHR-20-00230 33105083

[B49] McCartney E. Forbes J. (2023). SLPs and education: social capital relations during Covid-19 disruption. Scott. Edu. Rev. 1, 1–22. 10.1163/27730840-20231004

[B50] McGregor K. K. (2020). How we fail children with developmental language disorder. Lang. Speech Hear. Serv. Sch. 51, 981–992. 10.1044/2020_LSHSS-20-00003 32755505 PMC7842848

[B51] McGregor K. K. Goffman L. Van Horne A. O. Hogan T. P. Finestack L. H. (2020). Developmental language disorder: applications for advocacy, research, and clinical service. Perspect. ASHA Spec. Interest Groups 5, 38–46. 10.1044/2019_PERSP-19-0008336515428

[B52] McGregor K. K. Smolak E. Jones M. Oleson J. Eden N. Arbisi-Kelm T. . (2022). What children with developmental Language Disorder teach us about cross-situational word learning. Cogn. Sci. 46:13094. 10.1111/cogs.13094 35122309 PMC9285947

[B53] Monaghan P. (2017). Canalization of language structure from environmental constraints: a computational model of word learning from multiple cues. Top. Cogn. Sci. 9, 21–34. 10.1111/tops.12239 27989019 PMC6849513

[B54] Motsch H. J. Marks D. K. (2015). Efficacy of the Lexicon Pirate strategy therapy for improving lexical learning in school-age children: a randomized controlled trial. Child Lang. Teach. Ther. 31, 237–255. 10.1177/0265659014564678

[B55] Nash M. Donaldson M. L. (2005). Word learning in children with vocabulary deficits. J. Speech Lang. Hear. Res. 48, 439–458. 10.1044/1092-4388(2005/030) 15989403

[B56] Newcomer P. L. Hammil D. D. (1997). Test of Language Development - Primary: Third Edition. Austin, TX: Pro-Ed.

[B57] Norbury C. F. Gooch D. Wray C. Baird G. Charman T. Simonoff E. . (2016). The impact of nonverbal ability on prevalence and clinical presentation of Language Disorder: evidence from a population study. J. Child Psychol. Psychiatry 57, 1247–1257. 10.1111/jcpp.12573 27184709 PMC5082564

[B58] Page M. J. McKenzie J. E. Bossuyt P. M. Boutron I. Hoffmann T. C. Mulrow C. D. . (2021). The PRISMA 2020 statement: an updated guideline for reporting systematic reviews. Br. Med. J. 372:n71. 10.1136/bmj.n71 33782057 PMC8005924

[B59] Parsons S. Law J. Gascoigne M. (2005). Teaching receptive vocabulary to children with specific language impairment: a curriculum-based approach. Child Lang. Teach. Ther. 21, 39–59. 10.1191/0265659005ct280oa

[B60] Patel R. Chiat S. Cartwright M. Herman R. Sells R. (2022). Efficacy of vocabulary interventions for children of primary-school-age (5-11 years) with Language Disorder: protocol for a systematic review. PROSPERO 2022 CRD42022327345 Available at: https://www.crd.york.ac.uk/prospero/display_record.php?ID=CRD42022327345 (accessed May 17, 2022).

[B61] Pérez J. Díaz J. Garcia-Martin J. Tabuenca B. (2020). Systematic literature reviews in software engineering. Enhancement of the study selection process using Cohen's K. J. Syst. Softw. 168:110657. 10.1016/j.jss.2020.110657

[B62] Petermann F. (2010). Sprachstandserhebungstest für Fünf- bis Zehnjährige (SET 5–10) [Test of language development for children between five and ten, SET 5–10]. Göttingen: Hogrefe. 10.1055/s-0031-1285861 22009297

[B63] Plaut D. C. (1999). “Connectionist approaches to word processing,” in Handbook of Psycholinguistics (ed) M. Gernsbacher (London: Academic Press Ltd), 877–905.

[B64] Pomper R. (2020). Combining Cues in Novel Word Learning. The University of Wisconsin-Madison (Doctoral dissertation). Available at: Combining Cues in Novel Word Learning-ProQuest.

[B65] Renfrew C. (1995). Word Finding Vocabulary Test (4th ed.). Bicester: Speechmark Publishing.

[B66] Rice M. L. Cleave P. L. Oetting J. B. (2000). The use of syntactic cues in lexical acquisition by children with SLI. J. Speech Lang. Hear. Res. 43, 582–594. 10.1044/jslhr.4303.582 10877430

[B67] Rice M. L. Hoffman L. (2015). Predicting vocabulary growth in children with and without specific language impairment:alongitudinal study from 2; 6 to 21 years of age. JSLHR 58, 345–359. 10.1044/2015_JSLHR-L-14-0150 25611623 PMC4398600

[B68] Rinaldi S. Caselli M. C. Cofelice V. D'Amico S. De Cagno A. G. Della Corte G. . (2021). Efficacy of the treatment of developmental Language Disorder: a systematic review. Brain Sci. 11:407. 10.3390/brainsci11030407 33806938 PMC8005159

[B69] Roberts M. Y. Kaiser A. P. (2011). The effectiveness of parent-implemented language interventions: a meta-analysis. Am. J. Speech-Lang. Pathol. 20, 180–199 10.1044/1058-0360(2011/10-0055) 21478280

[B70] Roembke T. C. Simonetti M. E. Koch I. Philipp A. M. (2023). What have we learned from 15 years of research on cross-situational word learning? A focused review. Front. Psychol. 14:1175272. 10.3389/fpsyg.2023.1175272 37546430 PMC10400455

[B71] Royal College of Speech and Language Therapists: RCSLT (2021). Giving Voice to People with DLD. Available at: https://www.rcslt.org/wp-content/uploads/2021/10/rcslt-dld-factsheet.pdf (accessed 20 May 2023).

[B72] Senter R. (2022). Speech-Language Pathologists' Services for Children with Co-Occurring Language and Executive Function Deficits Doctoral dissertation, University of Maryland. Available at: ProQuest Dissertations & Theses Global: www.proquest.com/dissertations-theses/speech-language-pathologists-services-children/docview/2688138491/se-2?accountid=10406 (accessed October 22, 2022).

[B73] Siedler M. (2022). Sink or SWiM? When and How to Use Narrative Synthesis in Lieu of Meta-Analysis. Available at: https://usblog.gradeworkinggroup.org/2020/05/ (accessed October 22, 2023).

[B74] Smeets D. J. Van Dijken M. J. Bus A. G. (2014). Using electronic storybooks to support word learning in children with severe language impairments. J. Learn. Disabil. 47, 435–449 10.1177/0022219412467069 23213051

[B75] Stahl S. A. Murray B. A. (1994). Defining phonological awareness and its relationship to early reading. J. Educ. Psychol. 86, 221–234. 10.1037//0022-0663.86.2.221

[B76] Stark R. E. Tallal P. (1981). Selection of children with specific language deficits. J. Speech Hear. Disord. 46, 114–122. 10.1044/jshd.4602.114 7253588

[B77] Steele S. C. (2020). Vocabulary intervention: a national survey of school-based speech–language pathologists. Commun. Disord. Q. 41, 151–161. 10.1177/152574011982700838295301

[B78] Steele S. C. Mills M. T. (2011). Vocabulary intervention for school-age children with language impairment: a review of evidence and good practice. Child Lang. Teach. Ther. 27, 354–370. 10.1177/0265659011412247 25104872 PMC4122318

[B79] Steele S. C. Willoughby L. M. Mills M. T. (2013). Learning word meanings during reading: effects of phonological and semantic cues on children with language impairment. Int. J. Speech Lang. Pathol. 15, 184–197. 10.3109/17549507.2012.700322 22934530

[B80] Storkel H. L. Komesidou R. Fleming K. K. Romine R. S. (2017). Interactive book reading to accelerate word learning by kindergarten children with SLI: identifying adequate progress and successful learning patterns. Lang. Speech Hear. Serv. Schools 48, 108–124. 10.1044/2017_LSHSS-16-0058 28419188 PMC5544187

[B81] Storkel H. L. Komesidou R. Pezold M. J. Pitt A. R. Fleming K. K. Romine R. S. (2019). The impact of dose and dose frequency on word learning by kindergarten children with developmental Language Disorder during interactive book reading. Lang. Speech Hear. Serv. Sch. 50, 518–539. 10.1044/2019_LSHSS-VOIA-18-0131 31600474 PMC7210430

[B82] Stothard S. E. Snowling M. J. Bishop D. V. Chipchase B. B. Kaplan C. A. (1998). Language-impaired preschoolers: a follow-up into adolescence. J. Speech Lang. Hear. Res. 41, 407–418. 10.1044/jslhr.4102.407 9570592

[B83] Throneburg R. N. Calvert L. K. Sturm J. J. Paramboukas A. A. Paul P. J. (2000). A comparison of service delivery models: effects on curricular vocabulary skills in the school setting. Am. J. Speech-Lang. Pathol. 9, 10–20. 10.1044/1058-0360.0901.10

[B84] Tomblin J. B. Records N. L. Buckwalter P. Zhang X. Smith E. O'Brien M. (1997). Prevalence of specific language impairment in kindergarten children. JSLHR 40, 1245–1260. 10.1044/jslhr.4006.1245 9430746 PMC5075245

[B85] Trueswell J. C. Medina T. N. Hafri A. Gleitman L. R. (2013). Propose but verify: fast mapping meets cross-situational word learning. Cogn. Psychol. 66, 126–156. 10.1016/j.cogpsych.2012.10.001 23142693 PMC3529979

[B86] Tufanaru C. Munn Z. Aromataris E. Campbell J. Hopp L. (2020). “Systematic reviews of effectiveness,” in: *JBI Manual for Evidence Synthesis* (eds) E. Aromataris, C. Lockwood, K. Porritt, B. Pilla, and Z. Jordan (JBI). Available at: https://synthesismanual.jbi.global (accessed March 1, 2022).

[B87] Wanicharoen N. Boonrod V. (2024). Effect of music therapy on language skills in children with specific language impairment: a systematic review. J. Assoc. Med. Sci. 57, 96–103. 10.12982/JAMS.2024.011

[B88] Watt A. White S. (2018). Efficacy of group versus individual therapy for advancing receptive and expressive language development for children aged 6–12 years within community settings: a critically appraised topic. Evid. Based Commun. Assess. Interv. 12, 54–71. 10.1080/17489539.2018.1444914

[B89] Westrupp E. M. Reilly S. McKean C. Law J. Mensah F. Nicholson J. M. (2020). Vocabulary development and trajectories of behavioral and emotional difficulties via academic ability and peer problems. Child Dev. 91, e365–e382. 10.1111/cdev.13219 30697706

[B90] Wilson J. (2016). Data Collection Form for Intervention Reviews for RCTs and Non-RCTs - Template. The Cochrane Collaboration. Available at:https://dplp.cochrane.org/data-extraction-forms (accessed March 1, 2022).

[B91] Wing C. S. (1990). A preliminary investigation of generalization to untrained words following two treatments of children's word-finding problems. Lang. Speech Hear. Serv. Sch. 21, 151–156. 10.1044/0161-1461.2103.151

[B92] Wright S. H. (1993). Teaching word—finding strategies to severely language—impaired children. Eur. J. Disord. Commun. 28, 165–175. 10.3109/13682829309041464 8400488

[B93] Zens N. K. (2009). Doctoral Thesis: Facilitating word-learning abilities in children with specific language impairment. University of Canterbury. Available at: Microsoft Word - Naomi's Thesis examination editing.doc (canterbury.ac.nz).

[B94] Zens N. K. Gillon G. T. Moran C. (2009). Effects of phonological awareness and semantic intervention on word-learning in children with SLI. *Int. J. Speech-Lang. Pathol*. 11, 509–524. 21271927 10.3109/17549500902926881

[B95] Zupan B. Hutchings S. M. Everitt L. E. Gupta C. (2022). Language disorder and internalizing mental health problems in youth offenders: a systematic review. Int. J. Lang. Commun. Disord. 57, 1207–1212 10.1111/1460-6984.12759 35841339 PMC9796836

