# Supplementary material for: Vocabulary interventions for children with developmental language disorder: a systematic review
Source: Front Psychol. 2025 Mar 19;16:1517311. doi: 10.3389/fpsyg.2025.1517311 (PMC11962024; doi:10.3389/fpsyg.2025.1517311)
Supplement: Supplementary file 1 [file Supplementary_file_1.docx]

**Appendices**

**Appendix A: Quality Appraisal**

**Appendix A, Table 1: Studies appraised using the Joanna Briggs Institute critical appraisal checklist for randomised controlled trials.**

| **Study** | **Design** | **True randomisation** | **Allocation concealed** | **Similar groups at baseline** | **Participants blind to assignment** | **Providers blind to assignment** | **Treatment groups treated equally** | **Outcome assessors blind to assignment** | **Outcomes measured in same way** | **Outcomes measured reliably** | **Follow up** | **Participants analysed in groups randomised** | **Appropriate statistical analysis** | **Trial design appropriate** | **Rating based on no of Y:**  **≤49% low,**  **50-79% medium,**  **≥80% high** | | |  |
| --- | --- | --- | --- | --- | --- | --- | --- | --- | --- | --- | --- | --- | --- | --- | --- | --- | --- | --- |
| **Best et al. (2018)** | No-treatment control group | Y | Y | Y | N/A | N/A | N/A | Y | Y | Y | N | Y | Y | N^a^ | 82% | | **High** | |
| **Best et al. (2021)** | Alternating treatment crossover | Y | Y | Y | N/A | N/A | Y | Y | Y | Y | Y | Y | Y | Y | 100% | | **High** | |
| **Lowman & Dressler (2016)** | Alternating treatment crossover | Y | N | ?^b^ | N/A | N/A | ?^c^ | Y | Y | Y | N | Y | Y | N^a/d^ | 55% | | **Medium** | |
| **Motsch & Marks (2015)** | 2 treatment groups & 1 control group | Y | Y | Y | N/A | N/A | ?^c^ | Y | Y | Y | Y | Y | Y | Y | 82% | **High** | | |
| **Smeets et al. (2014) Study 1** | Alternating treatment crossover | Y | Y | Y | N/A | N/A | ?^b^ | Y | Y | Y | N | Y | N^e^ | N^d^ | 64% | **Medium** | | |
| **Smeets et al. (2014) Study 2** | Alternating treatment crossover | Y | Y | Y | N/A | N/A | ?^b^ | Y | Y | Y | N | Y | N^e^ | N^d^ | 64% | **Medium** | | |
| **Steele et al. (2013)** | Alternating treatment crossover | Y | N | Y | N/A | N/A | ?^b^ | Y | Y | Y | N | Y | N^e/f^ | N^a/d^ | 55% | | **Medium** | |
| **Storkel (2019)** | Alternating treatment crossover | Y | N | Y | N/A | N/A | Y | Y | Y | Y | Y | Y | N^f^ | Y | 82% | | **High** | |
| **Zens et al. (2009)** | Alternating treatment crossover | Y | N | Y | N/A | N/A | ?^b^ | N | Y | Y | N | Y | Y | N^a/d^ | 55% | | **Medium** | |

^a^ Small sample size (n < 20)

^b^ Gender unknown

^c^ Ambiguity regarding access to ongoing specialist language support in addition to experimental intervention

^d^ No time break between treatment crossovers

^e^ No order effect analysis

^f^ Effect size not reported

**Appendix A, Table 2: Studies appraised using the**

| **Study** | **Design** | **Clarity between cause and effect** | **Similar participant characteristics** | **Similar treatment for each participant** | **Control group** | **Multiple measure of outcome** | **Follow up** | **Outcomes measured consistently** | **Outcomes measured reliably** | **Appropriate statistical analysis** | **Rating based on no of Y:**  **≤49% low,**  **50-79% medium,**  **≥80% high** | |
| --- | --- | --- | --- | --- | --- | --- | --- | --- | --- | --- | --- | --- |
| **Ardanouy et al. (2023)** | Within group comparison | Y | ?^a^ | Y | N | Y | Y | Y | Y | N^b^ | 67% | **Medium** |
| **Best (2005)** | Case series | N^c^ | N | ?^c^ | N | Y | Y | N | Y | N^b/d^ | 33% | **Low** |
| [**Wright**](https://pubs-asha-org.eu1.proxy.openathens.net/doi/10.1044/0161-1461(2008/012)#bib56) **(1993)** | Matched no-treatment control | N^c^ | Y | ? ^c^ | Y | Y | Y | Y | Y | N^b/d^ | 67% | **Medium** |
| **Marks & Stokes (2010)** | Case study | Y | N/A | N/A | N | Y | Y | Y | Y | N^b/d^ | 71% | **Medium** |
| **Nash & Donaldson (2005)** | Alternating treatment crossover (one week washout) | N^c/e^ | Y | ? ^c^ | Y | Y | N | Y | Y | N^b/d^ | 56% | **Medium** |
| **Parsons et al. (2005)** | Case series | Y | Y | Y | N | N | N | Y | Y | N^b/d^ | 56% | **Medium** |
| **Wing (1990)** | Matched group comparison | N^c^ | Y | Y | N | N | N | Y | Y | N^b^ | 44% | **Low** |

^a^ Gender unknown

^b^ Small sample size (all studies had less than 20 participants per group)

^c^ Ambiguity regarding access to ongoing specialist language support in addition to experimental intervention

^d^ Effect size not reported

^e^ No order effect analysis
